# A hybrid particle swarm optimization algorithm for solving engineering problem

**DOI:** 10.1038/s41598-024-59034-2

**Published:** 2024-04-10

**Authors:** Jinwei Qiao, Guangyuan Wang, Zhi Yang, Xiaochuan Luo, Jun Chen, Kan Li, Pengbo Liu

**Affiliations:** 1 School of Mechanical and Automotive Engineering, Qilu University of Technology (Shandong Academy of Sciences), Jinan, 250353 China; 2 Shandong Institute of Mechanical Design and Research, Jinan, 250353 China; 3 School of Information Science and Engineering, Northeastern University, Shenyang, 110819 China; 4 Fushun Supervision Inspection Institute for Special Equipment, Fushun, 113000 China

**Keywords:** Particle swarm optimization, Elite opposition-based learning, Iterative mapping, Convergence analysis, Mechanical engineering, Computational science

## Abstract

To overcome the disadvantages of premature convergence and easy trapping into local optimum solutions, this paper proposes an improved particle swarm optimization algorithm (named NDWPSO algorithm) based on multiple hybrid strategies. Firstly, the elite opposition-based learning method is utilized to initialize the particle position matrix. Secondly, the dynamic inertial weight parameters are given to improve the global search speed in the early iterative phase. Thirdly, a new local optimal jump-out strategy is proposed to overcome the "premature" problem. Finally, the algorithm applies the spiral shrinkage search strategy from the whale optimization algorithm (WOA) and the Differential Evolution (DE) mutation strategy in the later iteration to accelerate the convergence speed. The NDWPSO is further compared with other 8 well-known nature-inspired algorithms (3 PSO variants and 5 other intelligent algorithms) on 23 benchmark test functions and three practical engineering problems. Simulation results prove that the NDWPSO algorithm obtains better results for all 49 sets of data than the other 3 PSO variants. Compared with 5 other intelligent algorithms, the NDWPSO obtains 69.2%, 84.6%, and 84.6% of the best results for the benchmark function (${f}_{1}-{f}_{13}$) with 3 kinds of dimensional spaces (Dim = 30,50,100) and 80% of the best optimal solutions for 10 fixed-multimodal benchmark functions. Also, the best design solutions are obtained by NDWPSO for all 3 classical practical engineering problems.

## Introduction

In the ever-changing society, new optimization problems arise every moment, and they are distributed in various fields, such as automation control^1^, statistical physics^2^, security prevention and temperature prediction^3^, artificial intelligence^4^, and telecommunication technology^5^. Faced with a constant stream of practical engineering optimization problems, traditional solution methods gradually lose their efficiency and convenience, making it more and more expensive to solve the problems. Therefore, researchers have developed many metaheuristic algorithms and successfully applied them to the solution of optimization problems. Among them, Particle swarm optimization (PSO) algorithm^6^ is one of the most widely used swarm intelligence algorithms.

However, the basic PSO has a simple operating principle and solves problems with high efficiency and good computational performance, but it suffers from the disadvantages of easily trapping in local optima and premature convergence. To improve the overall performance of the particle swarm algorithm, an improved particle swarm optimization algorithm is proposed by the multiple hybrid strategy in this paper. The improved PSO incorporates the search ideas of other intelligent algorithms (DE, WOA), so the improved algorithm proposed in this paper is named NDWPSO. The main improvement schemes are divided into the following 4 points: Firstly, a strategy of elite opposition-based learning is introduced into the particle population position initialization. A high-quality initialization matrix of population position can improve the convergence speed of the algorithm. Secondly, a dynamic weight methodology is adopted for the acceleration coefficients by combining the iterative map and linearly transformed method. This method utilizes the chaotic nature of the mapping function, the fast convergence capability of the dynamic weighting scheme, and the time-varying property of the acceleration coefficients. Thus, the global search and local search of the algorithm are balanced and the global search speed of the population is improved. Thirdly, a determination mechanism is set up to detect whether the algorithm falls into a local optimum. When the algorithm is “premature”, the population resets 40% of the position information to overcome the local optimum. Finally, the spiral shrinking mechanism combined with the DE/best/2 position mutation is used in the later iteration, which further improves the solution accuracy.

The structure of the paper is given as follows: Sect. “Particle swarm optimization (PSO)” describes the principle of the particle swarm algorithm. Section “Improved particle swarm optimization algorithm” shows the detailed improvement strategy and a comparison experiment of inertia weight is set up for the proposed NDWPSO. Section “Experiment and discussion” includes the experimental and result discussion sections on the performance of the improved algorithm. Section “Conclusions and future works” summarizes the main findings of this study.

## Literature review

This section reviews some metaheuristic algorithms and other improved PSO algorithms. A simple discussion about recently proposed research studies is given.

### Metaheuristic algorithms

A series of metaheuristic algorithms have been proposed in recent years by using various innovative approaches. For instance, Lin et al.^7^ proposed a novel artificial bee colony algorithm (ABCLGII) in 2018 and compared ABCLGII with other outstanding ABC variants on 52 frequently used test functions. Abed-alguni et al.^8^ proposed an exploratory cuckoo search (ECS) algorithm in 2021 and carried out several experiments to investigate the performance of ECS by 14 benchmark functions. Brajević^9^ presented a novel shuffle-based artificial bee colony (SB-ABC) algorithm for solving integer programming and minimax problems in 2021. The experiments are tested on 7 integer programming problems and 10 minimax problems. In 2022, Khan et al.^10^ proposed a non-deterministic meta-heuristic algorithm called Non-linear Activated Beetle Antennae Search (NABAS) for a non-convex tax-aware portfolio selection problem. Brajević et al.^11^ proposed a hybridization of the sine cosine algorithm (HSCA) in 2022 to solve 15 complex structural and mechanical engineering design optimization problems. Abed-Alguni et al.^12^ proposed an improved Salp Swarm Algorithm (ISSA) in 2022 for single-objective continuous optimization problems. A set of 14 standard benchmark functions was used to evaluate the performance of ISSA. In 2023, Nadimi et al.^13^ proposed a binary starling murmuration optimization (BSMO) to select the effective features from different important diseases. In the same year, Nadimi et al.^14^ systematically reviewed the last 5 years' developments of WOA and made a critical analysis of those WOA variants. In 2024, Fatahi et al.^15^ proposed an Improved Binary Quantum-based Avian Navigation Optimizer Algorithm (IBQANA) for the Feature Subset Selection problem in the medical area. Experimental evaluation on 12 medical datasets demonstrates that IBQANA outperforms 7 established algorithms. Abed-alguni et al.^16^ proposed an Improved Binary DJaya Algorithm (IBJA) to solve the Feature Selection problem in 2024. The IBJA’s performance was compared against 4 ML classifiers and 10 efficient optimization algorithms.

### Improved PSO algorithms

Many researchers have constantly proposed some improved PSO algorithms to solve engineering problems in different fields. For instance, Yeh^17^ proposed an improved particle swarm algorithm, which combines a new self-boundary search and a bivariate update mechanism, to solve the reliability redundancy allocation problem (RRAP) problem. Solomon et al.^18^ designed a collaborative multi-group particle swarm algorithm with high parallelism that was used to test the adaptability of Graphics Processing Units (GPUs) in distributed computing environments. Mukhopadhyay and Banerjee^19^ proposed a chaotic multi-group particle swarm optimization (CMS-PSO) to estimate the unknown parameters of an autonomous chaotic laser system. Duan et al.^20^ designed an improved particle swarm algorithm with nonlinear adjustment of inertia weights to improve the coupling accuracy between laser diodes and single-mode fibers. Sun et al.^21^ proposed a particle swarm optimization algorithm combined with non-Gaussian stochastic distribution for the optimal design of wind turbine blades. Based on a multiple swarm scheme, Liu et al.^22^ proposed an improved particle swarm optimization algorithm to predict the temperatures of steel billets for the reheating furnace. In 2022, Gad^23^ analyzed the existing 2140 papers on Swarm Intelligence between 2017 and 2019 and pointed out that the PSO algorithm still needs further research. In general, the improved methods can be classified into four categories:

1. Adjusting the distribution of algorithm parameters. Feng et al.^24^ used a nonlinear adaptive method on inertia weights to balance local and global search and introduced asynchronously varying acceleration coefficients.
2. Changing the updating formula of the particle swarm position. Both papers^25^ and^26^ used chaotic mapping functions to update the inertia weight parameters and combined them with a dynamic weighting strategy to update the particle swarm positions. This improved approach enables the particle swarm algorithm to be equipped with fast convergence of performance.
3. The initialization of the swarm. Alsaidy and Abbood proposed^27^ a hybrid task scheduling algorithm that replaced the random initialization of the meta-heuristic algorithm with the heuristic algorithms MCT-PSO and LJFP-PSO.
4. Combining with other intelligent algorithms: Liu et al.^28^ introduced the differential evolution (DE) algorithm into PSO to increase the particle swarm as diversity and reduce the probability of the population falling into local optimum.

## Particle swarm optimization (PSO)

The particle swarm optimization algorithm is a population intelligence algorithm for solving continuous and discrete optimization problems. It originated from the social behavior of individuals in bird and fish flocks^6^. The core of the PSO algorithm is that an individual particle identifies potential solutions by flight in a defined constraint space adjusts its exploration direction to approach the global optimal solution based on the shared information among the group, and finally solves the optimization problem. Each particle $i$ includes two attributes: velocity vector ${V}_{i}=\left[{v}_{i1},{v}_{i2},{v}_{i3},{...,v}_{ij},{...,v}_{iD},\right]$ and position vector ${X}_{i}=[{x}_{i1},{x}_{i2},{x}_{i3},...,{x}_{ij},...,{x}_{iD}]$. The velocity vector is used to modify the motion path of the swarm; the position vector represents a potential solution for the optimization problem. Here, $j=\mathrm{1,2},\dots ,D$, $D$ represents the dimension of the constraint space. The equations for updating the velocity and position of the particle swarm are shown in Eqs. (1) and (2).

1 $$v_{{{\text{ij}}}} \left( {k + 1} \right) = \omega \times v_{ij} \left( k \right) + r_{1} \times c_{1} \times \left( {Pbest_{i}^{k} - x_{ij} \left( k \right)} \right) + r_{2} \times c_{2} \times \left( {Gbest - x_{ij} \left( k \right)} \right)$$

2 $$x_{ij} \left( {k + 1} \right) = x_{ij} \left( k \right) + v_{ij} \left( {k + 1} \right)$$

Here ${Pbest}_{i}^{k}$ represents the previous optimal position of the particle $i$, and ${Gbest}$ is the optimal position discovered by the whole population. $i=\mathrm{1,2},\dots ,n$, $n$ denotes the size of the particle swarm. ${c}_{1}$ and ${c}_{2}$ are the acceleration constants, which are used to adjust the search step of the particle^29^. ${r}_{1}$ and ${r}_{2}$ are two random uniform values distributed in the range $[\mathrm{0,1}]$, which are used to improve the randomness of the particle search. $\omega$ inertia weight parameter, which is used to adjust the scale of the search range of the particle swarm^30^. The basic PSO sets the inertia weight parameter as a time-varying parameter to balance global exploration and local seeking. The updated equation of the inertia weight parameter is given as follows:

3 $$\omega = \omega_{max} - k \times \left( {\omega_{max} - \omega_{min} } \right)/Mk$$

where ${\omega }_{max}$ and ${\omega }_{min}$ represent the upper and lower limits of the range of inertia weight parameter. $k$ and $Mk$ are the current iteration and maximum iteration.

## Improved particle swarm optimization algorithm

According to the no free lunch theory^31^, it is known that no algorithm can solve every practical problem with high quality and efficiency for increasingly complex and diverse optimization problems. In this section, several improvement strategies are proposed to improve the search efficiency and overcome this shortcoming of the basic PSO algorithm.

### Improvement strategies

The optimization strategies of the improved PSO algorithm are shown as follows:

1. **The inertia weight parameter** is updated by an improved chaotic variables method instead of a linear decreasing strategy. Chaotic mapping performs the whole search at a higher speed and is more resistant to falling into local optimal than the probability-dependent random search^32^. However, the population may result in that particles can easily fly out of the global optimum boundary. To ensure that the population can converge to the global optimum, an improved Iterative mapping is adopted and shown as follows:
  4 $$\omega_{k + 1} = {\text{sin}}\left( {b \times \pi /\omega_{k} } \right) \times k/Mk$$
  Here ${\omega }_{k}$ is the inertia weight parameter in the iteration $k$, $b$ is the control parameter in the range $[\mathrm{0,1}]$.
2. **The acceleration coefficients** are updated by the linear transformation.${c}_{1}$ and ${c}_{2}$ represent the influential coefficients of the particles by their own and population information, respectively. To improve the search performance of the population, ${c}_{1}$ and ${c}_{2}$ are changed from fixed values to time-varying parameter parameters, that are updated by linear transformation with the number of iterations:
  5 $$c_{1} = c_{max} - \left( {c_{max} - c_{min} } \right) \times k/Mk$$
  6 $$c_{2} = c_{min} + \left( {c_{max} - c_{min} } \right) \times k/Mk$$
  where ${c}_{max}$ and ${c}_{min}$ are the maximum and minimum values of acceleration coefficients, respectively.
3. **The initialization scheme** is determined by elite opposition-based learning**.** The high-quality initial population will accelerate the solution speed of the algorithm and improve the accuracy of the optimal solution. Thus, the elite backward learning strategy^33^ is introduced to generate the position matrix of the initial population. Suppose the elite individual of the population is ${X}=[{x}_{1},{x}_{2},{x}_{3},...,{x}_{j},...,{x}_{D}]$, and the elite opposition-based solution of $X$ is ${X}_{o}=[{x}_{{\text{o}}1},{x}_{{\text{o}}2},{x}_{{\text{o}}3},...,{x}_{oj},...,{x}_{oD}]$. The formula for the elite opposition-based solution is as follows:
  7 $$x_{oij} = k_{r} \times \left( {ux_{oij} + lx_{oij} } \right) - x_{ij}$$
  8 $$ux_{oij} = {\text{max}}\left( {x_{ij} } \right) ,lx_{oij} = {\text{min}}\left( {x_{ij} } \right)$$
  where ${k}_{r}$ is the random value in the range $(\mathrm{0,1})$. ${ux}_{oij}$ and ${lx}_{oij}$ are dynamic boundaries of the elite opposition-based solution in $j$ dimensional variables. The advantage of dynamic boundary is to reduce the exploration space of particles, which is beneficial to the convergence of the algorithm. When the elite opposition-based solution is out of bounds, the out-of-bounds processing is performed. The equation is given as follows:
  9 $$x_{oij} = rand\left( {lx_{oij} ,ux_{oij} } \right)$$
  After calculating the fitness function values of the elite solution and the elite opposition-based solution, respectively, $n$ high quality solutions were selected to form a new initial population position matrix.
4. **The position updating **Eq. (2) is modified based on the strategy of dynamic weight. To improve the speed of the global search of the population, the strategy of dynamic weight from the artificial bee colony algorithm^34^ is introduced to enhance the computational performance. The new position updating equation is shown as follows:
  10 $$x_{ij} \left( {k + 1} \right) = \omega_{ } \times x_{ij} \left( k \right) + \omega^{\prime} \times v_{ij} \left( {k + 1} \right) + \rho \times \psi \times Gbest$$
  Here $\rho$ is the random value in the range $(\mathrm{0,1})$. $\psi$ represents the acceleration coefficient and ${\omega }{\prime}$ is the dynamic weight coefficient. The updated equations of the above parameters are as follows:
  11 $$\psi = \exp \left( {f\left( i \right)/u} \right)/\left( {1 + {\text{exp}}\left( { - f\left( i \right)/u} \right)} \right)^{iter}$$
  12 $$\omega^{\prime} = 1 - \omega_{ }$$
  where $f(i)$ denotes the fitness function value of individual particle $i$ and u is the average of the population fitness function values in the current iteration. The Eqs. (11,12) are introduced into the position updating equation. And they can attract the particle towards positions of the best-so-far solution in the search space.
5. **New local optimal jump-out strategy** is added for escaping from the local optimal. When the value of the fitness function for the population optimal particles does not change in M iterations, the algorithm determines that the population falls into a local optimal. The scheme in which the population jumps out of the local optimum is to reset the position information of the 40% of individuals within the population, in other words, to randomly generate the position vector in the search space. M is set to 5% of the maximum number of iterations.
6. **New spiral update search strategy** is added after the local optimal jump-out strategy. Since the whale optimization algorithm (WOA) was good at exploring the local search space^35^, the spiral update search strategy in the WOA^36^ is introduced to update the position of the particles after the swarm jumps out of local optimal. The equation for the spiral update is as follows:
  13 $$x_{ij} \left( {k + 1} \right) = D \times e^{B \times l} \times \cos \left( {2 \times pi \times l} \right) + Gbest$$
  Here $D=\left|{x}_{i}\left(k\right)-Gbest\right|$ denotes the distance between the particle itself and the global optimal solution so far. $B$ is the constant that defines the shape of the logarithmic spiral. $l$ is the random value in $[-\mathrm{1,1}]$.$l$ represents the distance between the newly generated particle and the global optimal position, $l=-1$ means the closest distance, while $l=1$ means the farthest distance, and the meaning of this parameter can be directly observed by Fig. 1.

**Figure 1:**
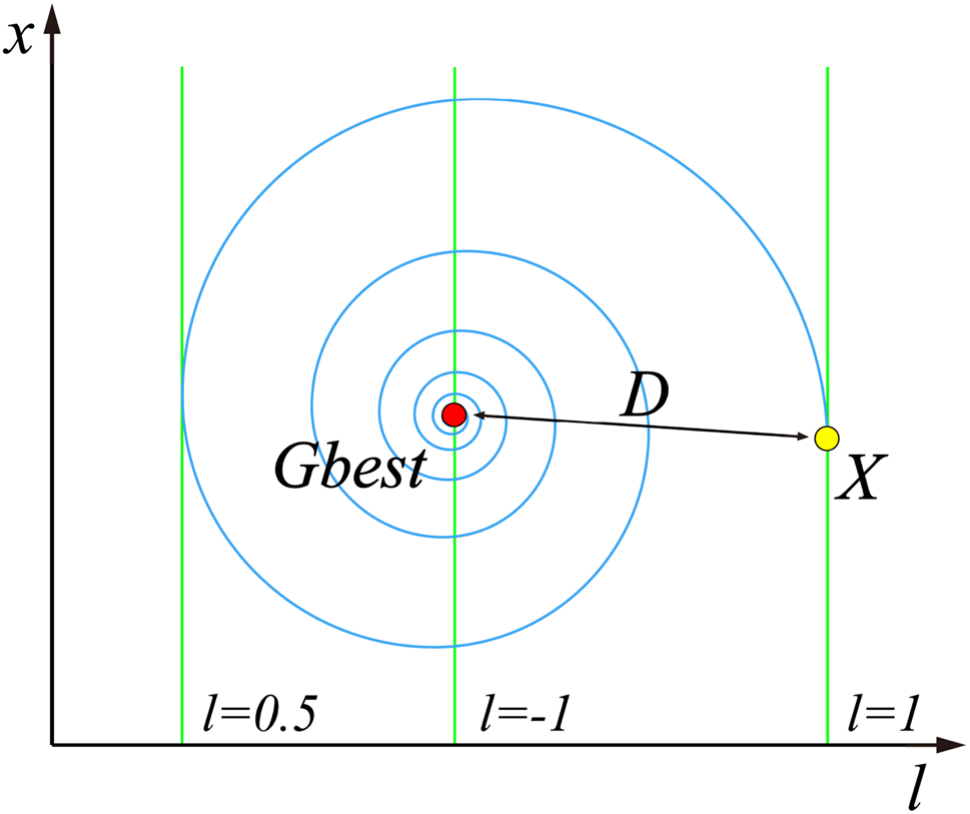
Spiral updating position.
7. **The DE/best/2 mutation strategy** is introduced to form the mutant particle. 4 individuals in the population are randomly selected that differ from the current particle, then the vector difference between them is rescaled, and the difference vector is combined with the global optimal position to form the mutant particle. The equation for mutation of particle position is shown as follows:
  14 $$x^{*} = Gbest + F \times \left( {x_{r1} - x_{r2} } \right) + F \times \left( {x_{r3} - x_{r4} } \right)$$
  where ${x}^{*}$ is the mutated particle, $F$ is the scale factor of mutation, ${r}_{1}$, ${r}_{2}$, ${r}_{3}$, ${r}_{4}$ are random integer values in $(0,n]$ and not equal to $i$, respectively. Specific particles are selected for mutation with the screening conditions as follows:
  15 $$x\left( {k + 1} \right) = \left\{ { \begin{array}{*{20}l} {x^{*} ,} \hfill & {if\;\left( {rand\left( {0,1} \right) < Cr\, or\, i = = i_{rand} } \right)} \hfill \\ {x\left( {k + 1} \right),} \hfill & { otherwise} \hfill \\ \end{array} } \right.$$
  where $Cr$ represents the probability of mutation, $rand\left(\mathrm{0,1}\right)$ is a random number in $\left(\mathrm{0,1}\right)$, and ${i}_{rand}$ is a random integer value in $(0,n]$.
  The improved PSO incorporates the search ideas of other intelligent algorithms (DE, WOA), so the improved algorithm proposed in this paper is named NDWPSO. The pseudo-code for the NDWPSO algorithm is given as follows:

**Algorithm 1:**
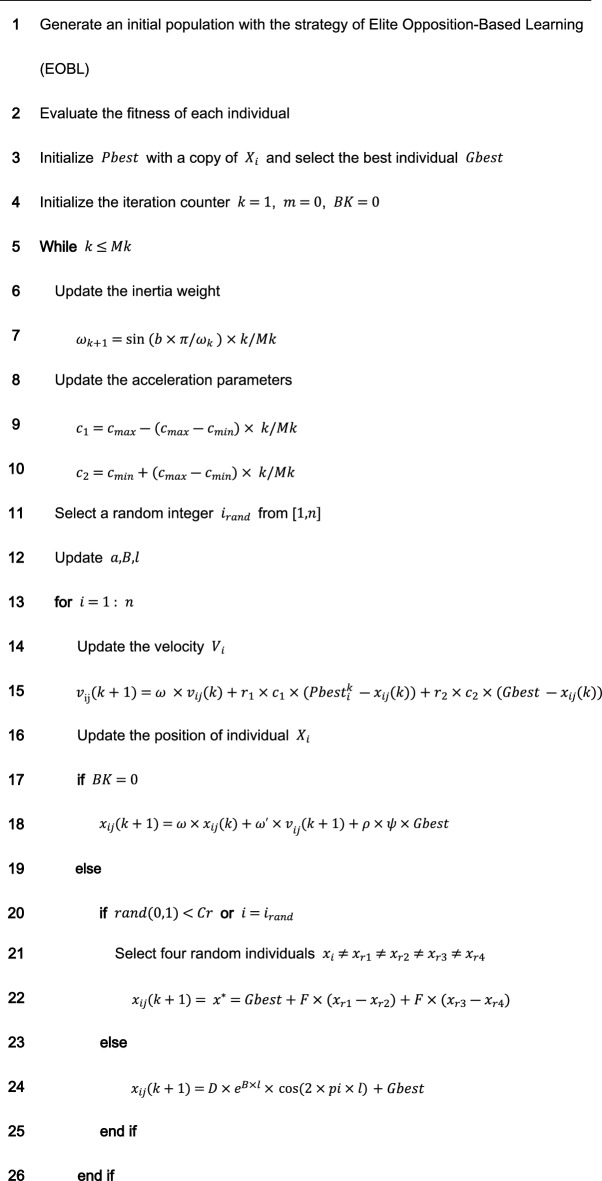

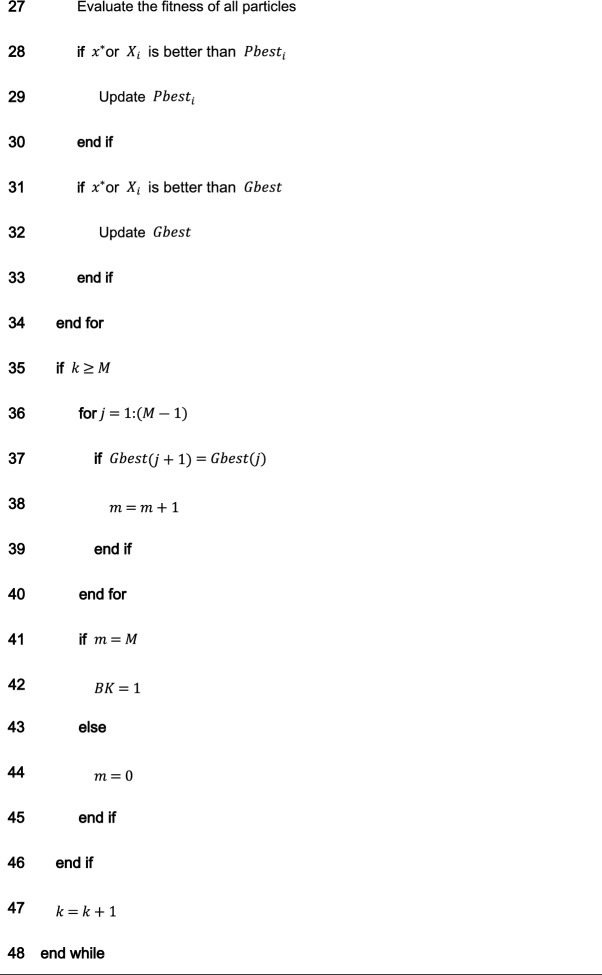
The main procedure of NDWPSO.

### Comparing the distribution of inertia weight parameters

There are several improved PSO algorithms (such as CDWPSO^25^, and SDWPSO^26^) that adopt the dynamic weighted particle position update strategy as their improvement strategy. The updated equations of the CDWPSO and the SDWPSO algorithm for the inertia weight parameters are given as follows:

16 $$\omega_{k + 1} = A \times {\text{sin}}\left( {\pi \times \omega_{k} } \right)$$

17 $$\omega_{k + 1} = \left( {r_{max} - \left( {r_{max} - r_{min} } \right)} \right)(k/Mk \times {\text{sin}}\left( {\pi \times \omega_{k} } \right)/4$$

where ${\text{A}}$ is a value in $(\mathrm{0,1}]$. ${r}_{max}$ and ${r}_{min}$ are the upper and lower limits of the fluctuation range of the inertia weight parameters, $k$ is the current number of algorithm iterations, and $Mk$ denotes the maximum number of iterations.

**Figure 2:**
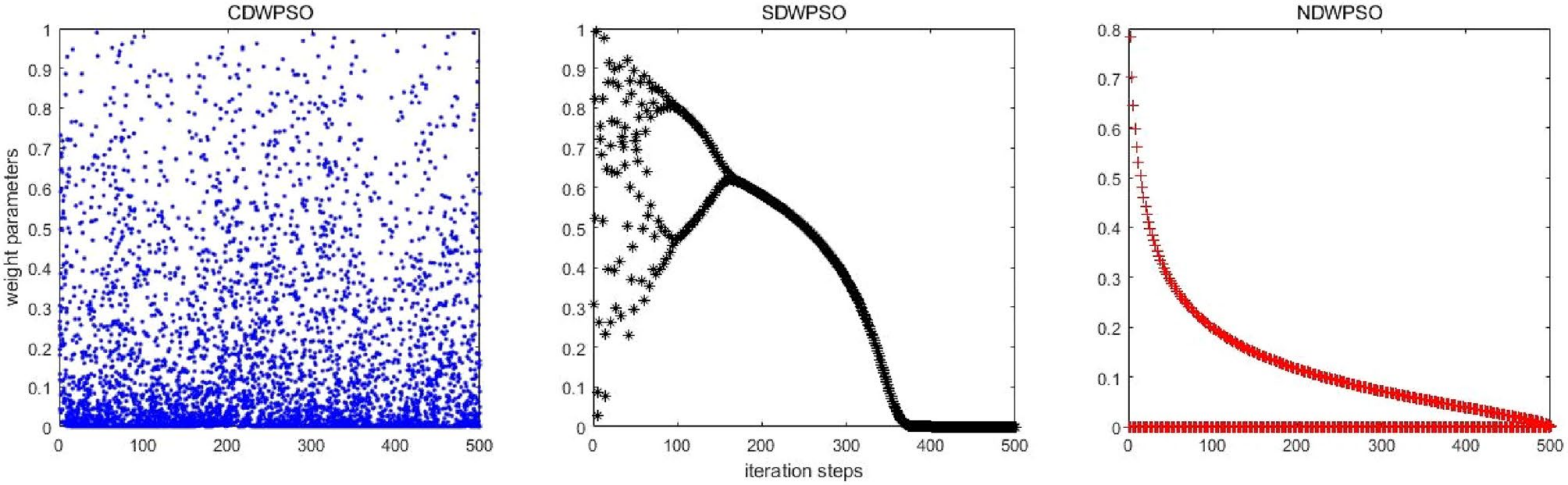
The inertial weight distribution of CDWPSO, SDWPSO, and NDWPSO.

Considering that the update method of inertia weight parameters by our proposed NDWPSO is comparable to the CDWPSO, and SDWPSO, a comparison experiment for the distribution of inertia weight parameters is set up in this section. The maximum number of iterations in the experiment is $Mk=500$. The distributions of CDWPSO, SDWPSO, and NDWPSO inertia weights are shown sequentially in Fig. 2.

In Fig. 2, the inertia weight value of CDWPSO is a random value in (0,1]. It may make individual particles fly out of the range in the late iteration of the algorithm. Similarly, the inertia weight value of SDWPSO is a value that tends to zero infinitely, so that the swarm no longer can fly in the search space, making the algorithm extremely easy to fall into the local optimal value. On the other hand, the distribution of the inertia weights of the NDWPSO forms a gentle slope by two curves. Thus, the swarm can faster lock the global optimum range in the early iterations and locate the global optimal more precisely in the late iterations. The reason is that the inertia weight values between two adjacent iterations are inversely proportional to each other. Besides, the time-varying part of the inertial weight within NDWPSO is designed to reduce the chaos characteristic of the parameters. The inertia weight value of NDWPSO avoids the disadvantages of the above two schemes, so its design is more reasonable.

## Experiment and discussion

In this section, three experiments are set up to evaluate the performance of NDWPSO: (1) the experiment of 23 classical functions^37^ between NDWPSO and three particle swarm algorithms (PSO^6^, CDWPSO^25^, SDWPSO^26^); (2) the experiment of benchmark test functions between NDWPSO and other intelligent algorithms (Whale Optimization Algorithm (WOA)^36^, Harris Hawk Algorithm (HHO)^38^, Gray Wolf Optimization Algorithm (GWO)^39^, Archimedes Algorithm (AOA)^40^, Equilibrium Optimizer (EO)^41^ and Differential Evolution (DE)^42^); (3) the experiment for solving three real engineering problems (welded beam design^43^, pressure vessel design^44^, and three-bar truss design^38^). All experiments are run on a computer with Intel i5-11400F GPU, 2.60 GHz, 16 GB RAM, and the code is written with MATLAB R2017b.

The benchmark test functions are 23 classical functions, which consist of indefinite unimodal (F1–F7), indefinite dimensional multimodal functions (F8–F13), and fixed-dimensional multimodal functions (F14–F23). The unimodal benchmark function is used to evaluate the global search performance of different algorithms, while the multimodal benchmark function reflects the ability of the algorithm to escape from the local optimal. The mathematical equations of the benchmark functions are shown and found as Supplementary Tables S1–S3 online.

### Experiments on benchmark functions between NDWPSO, and other PSO variants

The purpose of the experiment is to show the performance advantages of the NDWPSO algorithm. Here, the dimensions and corresponding population sizes of 13 benchmark functions (7 unimodal and 6 multimodal) are set to (30, 40), (50, 70), and (100, 130). The population size of 10 fixed multimodal functions is set to 40. Each algorithm is repeated 30 times independently, and the maximum number of iterations is 200. The performance of the algorithm is measured by the mean and the standard deviation (SD) of the results for different benchmark functions. The parameters of the NDWPSO are set as: ${[{\omega }_{min},\omega }_{max}]=[\mathrm{0.4,0.9}]$, $\left[{c}_{max},{c}_{min}\right]=\left[\mathrm{2.5,1.5}\right],{V}_{max}=0.1,b={e}^{-50}, M=0.05\times Mk, B=1,F=0.7, Cr=0.9.$ And, $A={\omega }_{max}$ for CDWPSO; ${[r}_{max},{r}_{min}]=[\mathrm{4,0}]$ for SDWPSO.

Besides, the experimental data are retained to two decimal places, but some experimental data will increase the number of retained data to pursue more accuracy in comparison. The best results in each group of experiments will be displayed in bold font. The experimental data is set to 0 if the value is below 10^–323^. The experimental parameter settings in this paper are different from the references (PSO^6^, CDWPSO^25^, SDWPSO^26^, so the final experimental data differ from the ones within the reference.

As shown in Tables 1 and 2, the NDWPSO algorithm obtains better results for all 49 sets of data than other PSO variants, which include not only 13 indefinite-dimensional benchmark functions and 10 fixed-multimodal benchmark functions. Remarkably, the SDWPSO algorithm obtains the same accuracy of calculation as NDWPSO for both unimodal functions f_1_–f_4_ and multimodal functions f_9_–f_11_. The solution accuracy of NDWPSO is higher than that of other PSO variants for fixed-multimodal benchmark functions f_14_-f_23_. The conclusion can be drawn that the NDWPSO has excellent global search capability, local search capability, and the capability for escaping the local optimal.

**Table 1:** Optimization results and comparison for functions (f_1_–f_13_).

Fun	Dim	CDWPSO		SDWPSO		PSO		NDWPSO	
		Ave	S.D	Ave	S.D	Ave	S.D	Ave	S.D
f_1_	30	2.06e−196	0	**0**	0	0.92	3.08	**0**	0
	50	1.37e−188	0	**0**	0	5.43e−02	0.13	**0**	0
	100	4.76e−199	0	**0**	0	5.78e−02	0.12	**0**	0
f_2_	30	2.51e−88	1.35e−87	**0**	0	2.96e−02	3.11e−02	**0**	0
	50	1.18e−95	4.81e−95	**0**	0	1.49e−02	1.70e−02	**0**	0
	100	1.36e−100	3.04e−100	**0**	0	8.72e−03	1.19e−02	**0**	0
f_3_	30	1.54e−178	0	**0**	0	4.42	6.36	**0**	0
	50	3.32e−187	0	**0**	0	2.53	4.79	**0**	0
	100	1.93e−199	0	**0**	0	1.36	3.61	**0**	0
f_4_	30	1.18e−84	4.68e−84	**0**	0	0.41	0.29	**0**	0
	50	1.83e−90	6.69e−90	**0**	0	0.3	0.24	**0**	0
	100	6.25e−97	3.12e−96	**0**	0	0.17	0.17	**0**	0
f_5_	30	2.87e + 01	3.08e−02	2.88e + 01	2.00e−02	3.45e + 01	1.20e + 01	**2.69e + 01**	0.64
	50	4.86e + 01	5.29e−02	4.88e + 01	2.58e−02	5.01e + 01	4.63	**4.62e + 01**	0.65
	100	9.85e + 01	9.29e−02	9.87e + 01	2.67e−02	9.85e + 01	0.35	**9.58e + 01**	0.66
f_6_	30	5.88	0.29	6.04	0.29	6.57	4.54	**0.14**	0.22
	50	9.93	0.46	1.03e + 01	0.45	6.29	2.73	**0.11**	8.83e−02
	100	2.07e + 01	0.42	2.13e + 01	0.65	1.06e + 01	4.14	**0.28**	0.18
f_7_	30	0.49	0.26	0.52	0.30	0.55	0.29	**0.39**	0.26
	50	0.57	0.29	0.45	0.28	0.57	0.28	**0.42**	0.30
	100	0.51	0.30	0.49	0.25	0.58	0.27	**0.47**	0.29
f_8_	30	−1335.64	8.64e + 02	−1872.55	1.22e + 03	−392.69	4.66e + 01	**−6418.01**	1.26e + 03
	50	−1813.72	7.83e + 02	−2634.55	1.24e + 03	−431.787	2.47e + 01	**−10,740.04**	2.50e + 03
	100	−2703.68	1.72e + 03	−3859.14	1.40e + 03	−457.21	3.34e + 01	**−20,964.07**	4.37e + 03
f_9_	30	**0**	0	**0**	0	6.92e−02	0.12	**0**	0
	50	**0**	0	**0**	0	4.64e−02	3.11e−02	**0**	0
	100	**0**	0	**0**	0	8.04e−03	1.50e−02	**0**	0
f_10_	30	**8.88e−16**	0	**8.88e−16**	0	4.75e−02	2.73e−02	**8.88e−16**	0
	50	**8.88e−16**	0	**8.88e−16**	0	3.01e−02	2.25e−02	**8.88e−16**	0
	100	**8.88e−16**	0	**8.88e−16**	0	1.54e−02	1.63e−02	**8.88e−16**	0
f_11_	30	**0**	0	**0**	0	0.26	0.41	**0**	0
	50	**0**	0	**0**	0	0.14	0.32	**0**	0
	100	**0**	0	**0**	0	2.86e−02	3.73e−02	**0**	0
f_12_	30	1.10	2.63e−02	1.10	6.73e−03	0.68	0.34	**4.58e−03**	1.93e−02
	50	1.12	2.06e−02	1.13	5.91e−03	0.43	0.32	**3.36e−03**	1.11e−02
	100	1.16	2.86e−02	1.15	4.44e−03	0.13	5.59e−02	**2.85e−03**	5.67e−03
f_13_	30	2.92	1.59e−02	2.91	6.36e−03	2.90	1.59e−02	**5.05e−02**	4.14e−02
	50	4.92	1.03e−02	4.91	5.94e−03	4.89	9.46e−03	**8.39e−02**	5.22e−02
	100	9.91	7.70e−03	9.91	4.93e−03	9.88	8.31e−03	**0.21**	0.11

Significant values in bold.

**Table 2:** Optimization results and comparison for functions (f_14_–f_23_).

Fun	Dim	CDWPSO		SDWPSO		PSO		NDWPSO	
		Ave	S.D	Ave	S.D	Ave	S.D	Ave	S.D
f_14_	2	1.08e + 01	0.12	1.08e + 01	1.01e−05	1.08e + 01	1.36e−02	**1.72**	2.43
f_15_	4	8.65e−03	5.87e−03	1.10e−02	8.58e−03	3.11e−04	2.10e−05	**3.07e−04**	1.17e−17
f_16_	2	−0.0402	1.97e−06	−0.0401	1.54e−04	**−1.0316**	4.71e−16	**−1.0316**	6.08e−16
f_17_	2	5.0437	4.07e−02	5.0255	3.57e−02	1.7906	2.13	**0.397**	0
f_18_	2	7.94e + 01	2.13e + 01	9.84e + 01	1.69e + 01	7.86e + 01	1.62e + 01	**3**	1.68e−15
f_19_	3	−2.4156	0.68	−2.8410	0.76	−1.0008	1.94e−16	**−3.86**	2.53e−15
f_20_	6	−1.09	0.22	−1.26	0.11	−3.25	0.23	**−3.27**	5.89e−02
f_21_	4	−1.08	1.39	−0.39	0.08	−3.04	2.15	**−7.13**	2.75
f_22_	4	−0.79	0.86	−0.39	1.73e−02	−2.81	2.14	**−8.01**	2.75
f_23_	4	−0.88	0.92	−0.43	2.31e−02	−3.01	2.12	**−7.83**	3.20

Significant values in bold.

**Figure 3:**
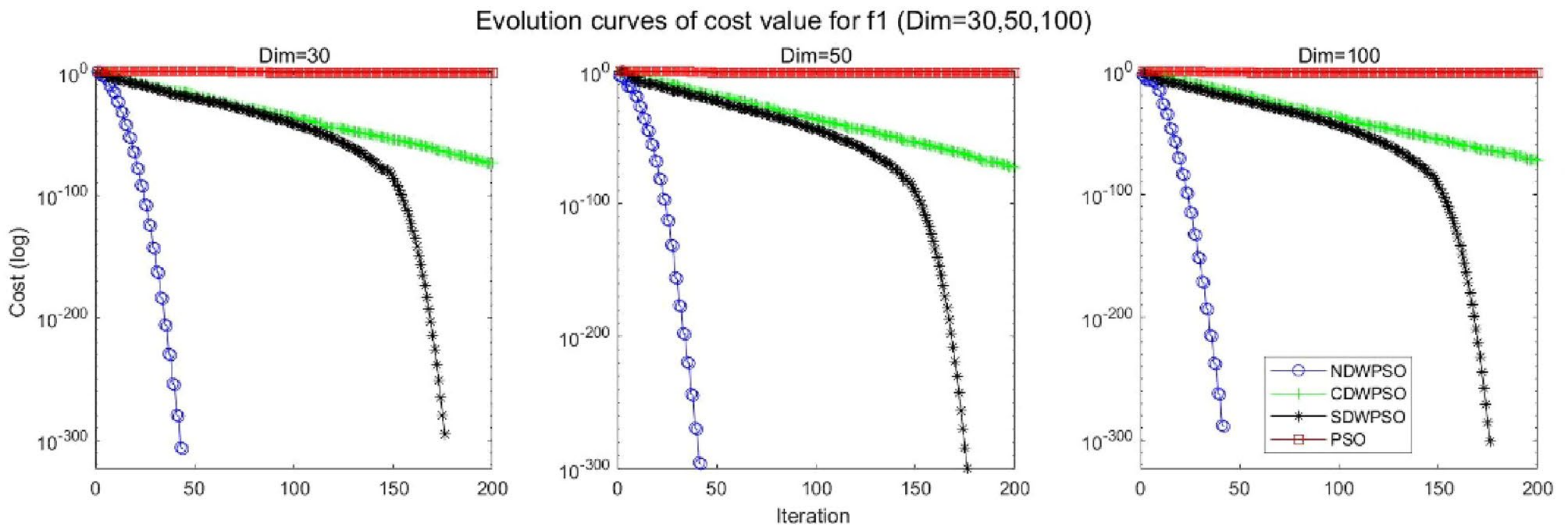
Evolution curve of NDWPSO and other PSO algorithms for f1 (Dim = 30,50,100).

**Figure 4:**
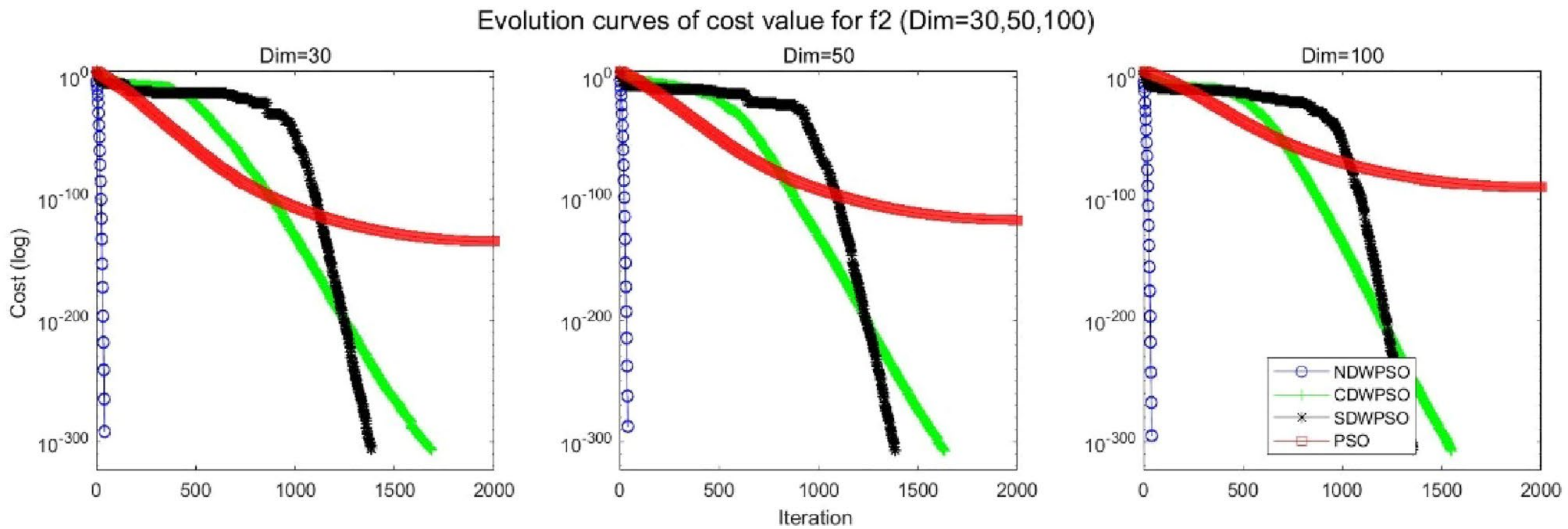
Evolution curve of NDWPSO and other PSO algorithms for f2 (Dim = 30,50,100).

**Figure 5:**
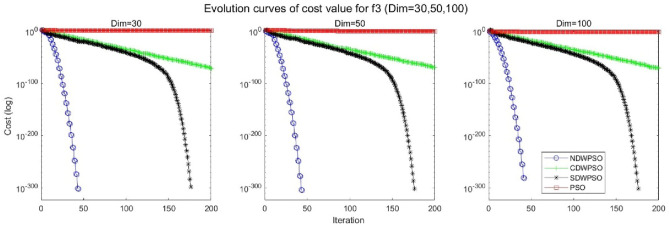
Evolution curve of NDWPSO and other PSO algorithms for f3 (Dim = 30,50,100).

**Figure 6:**
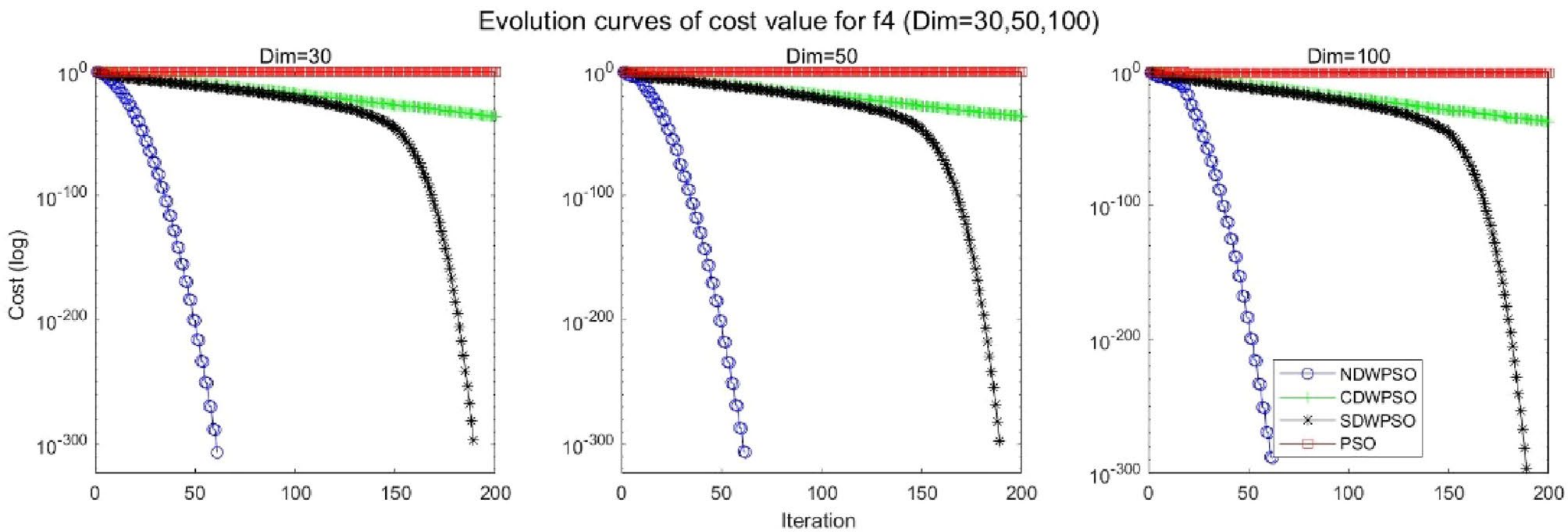
Evolution curve of NDWPSO and other PSO algorithms for f4 (Dim = 30,50,100).

**Figure 7:**
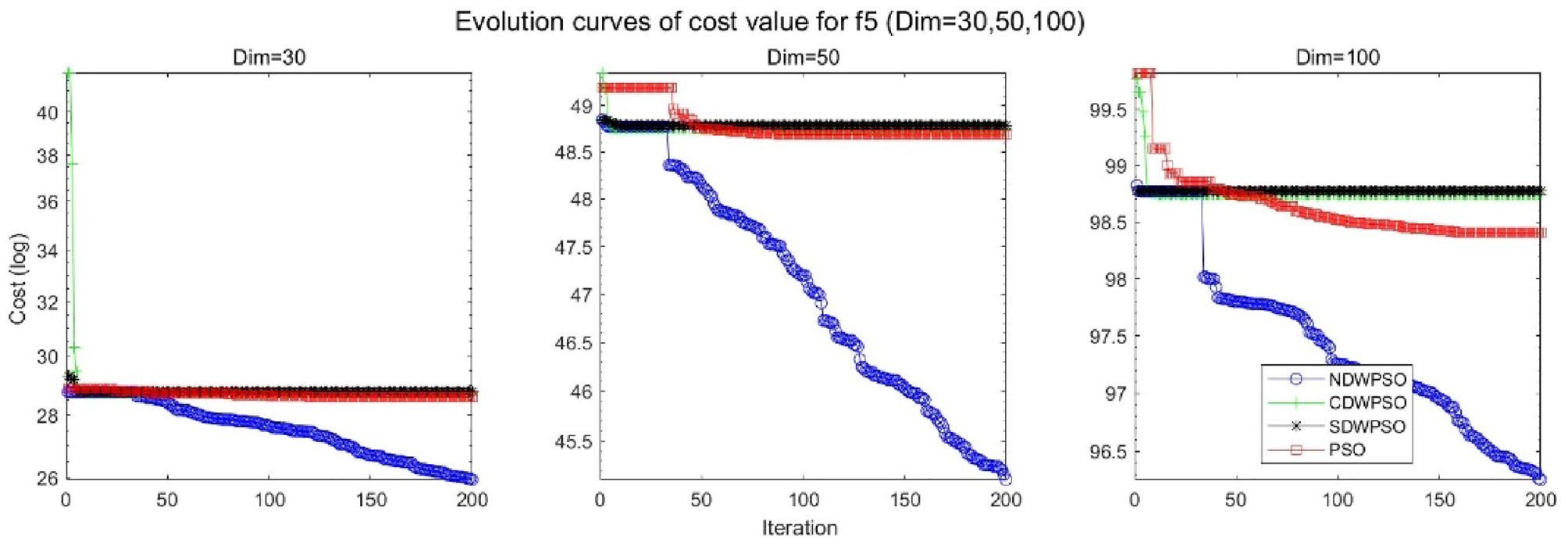
Evolution curve of NDWPSO and other PSO algorithms for f5 (Dim = 30,50,100).

**Figure 8:**
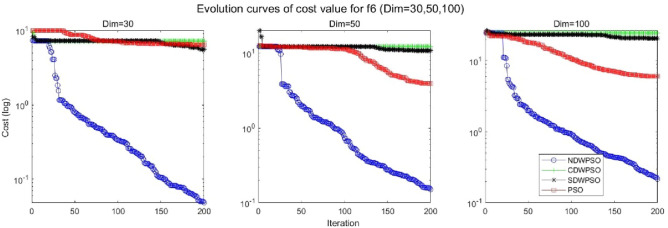
Evolution curve of NDWPSO and other PSO algorithms for f6 (Dim = 30,50,100).

**Figure 9:**
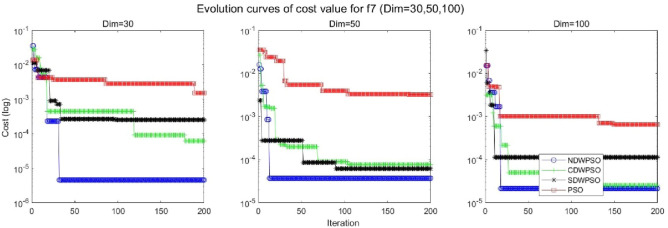
Evolution curve of NDWPSO and other PSO algorithms for f7 (Dim = 30,50,100).

**Figure 10:**
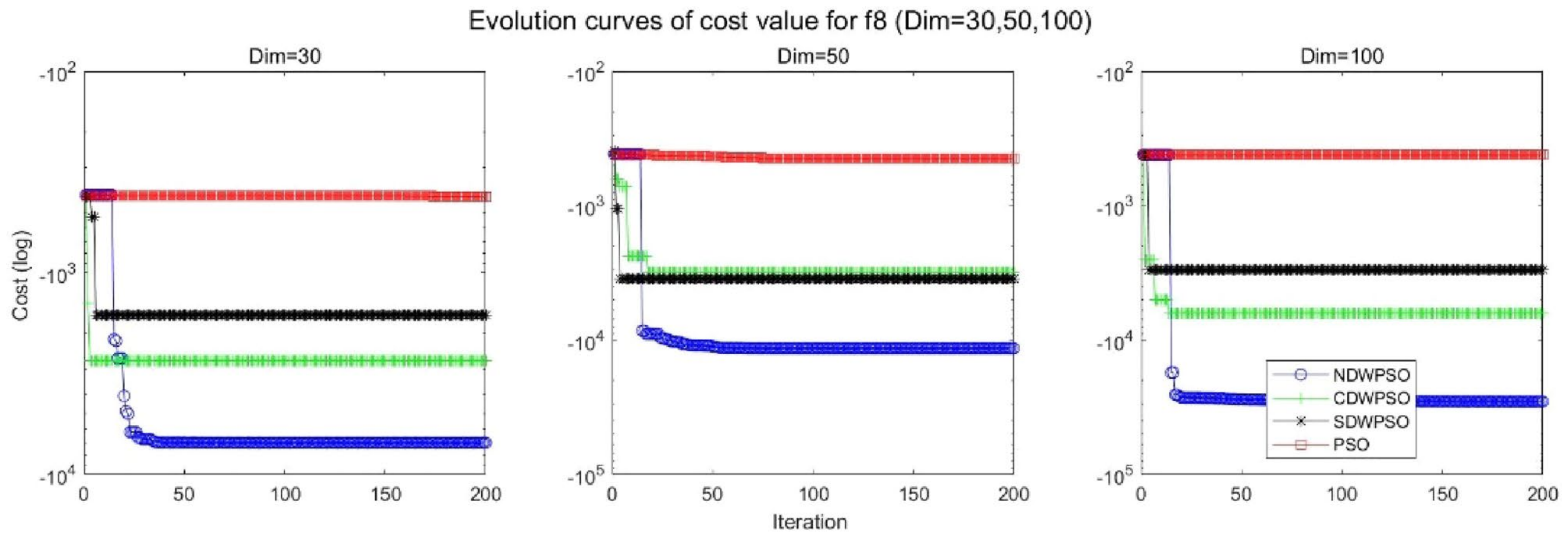
Evolution curve of NDWPSO and other PSO algorithms for f8 (Dim = 30,50,100).

**Figure 11:**
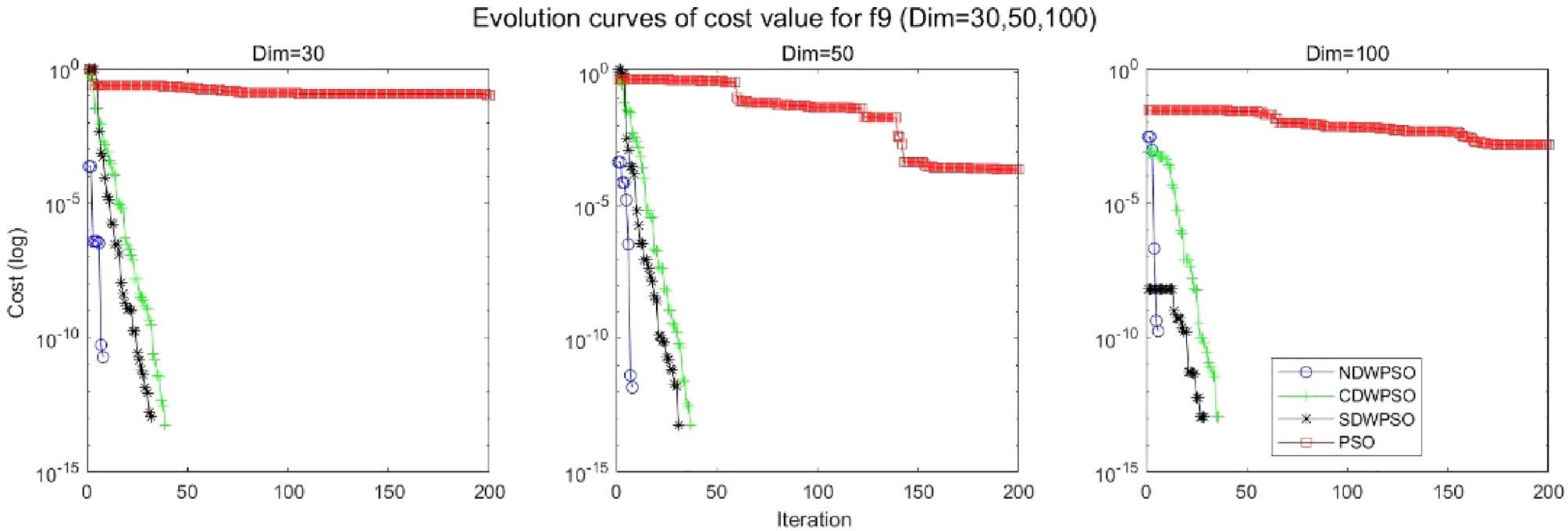
Evolution curve of NDWPSO and other PSO algorithms for f9 (Dim = 30,50,100).

**Figure 12:**
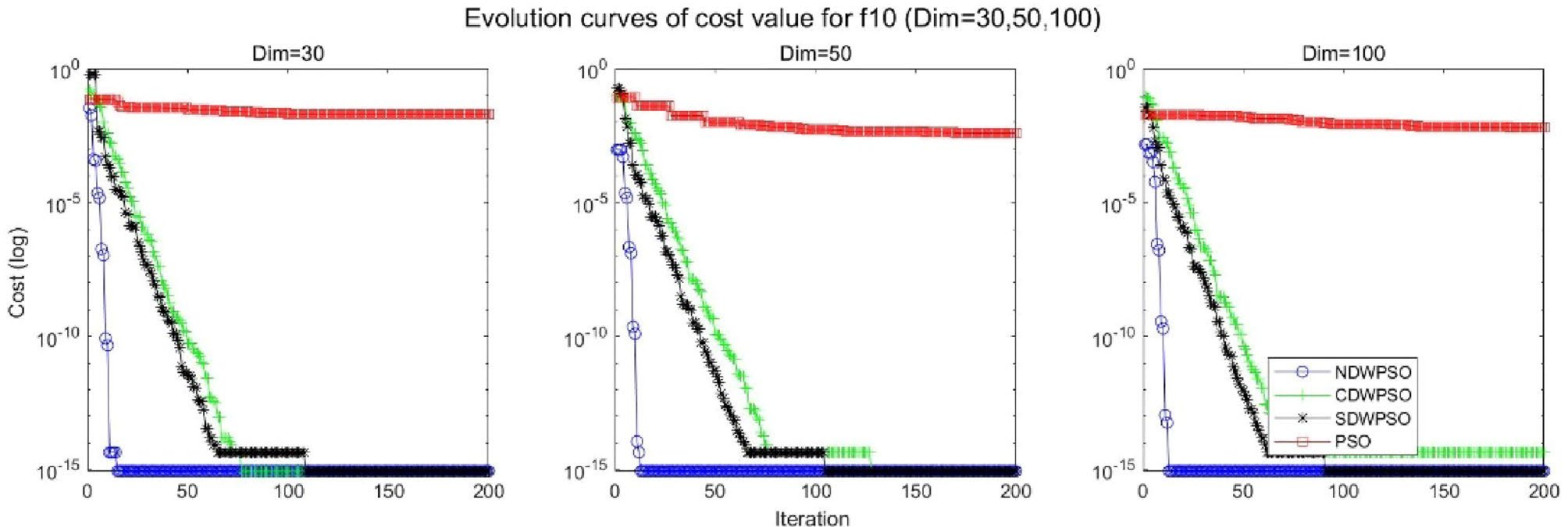
Evolution curve of NDWPSO and other PSO algorithms for f10 (Dim = 30,50,100).

**Figure 13:**
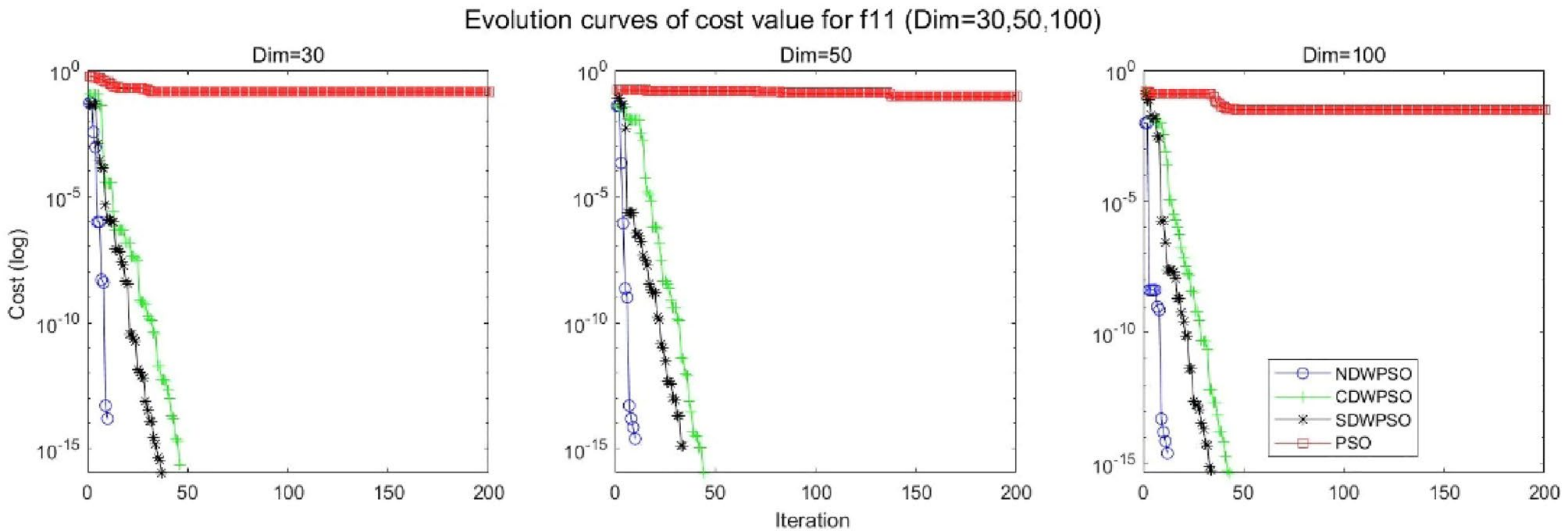
Evolution curve of NDWPSO and other PSO algorithms for f11(Dim = 30,50,100).

**Figure 14:**
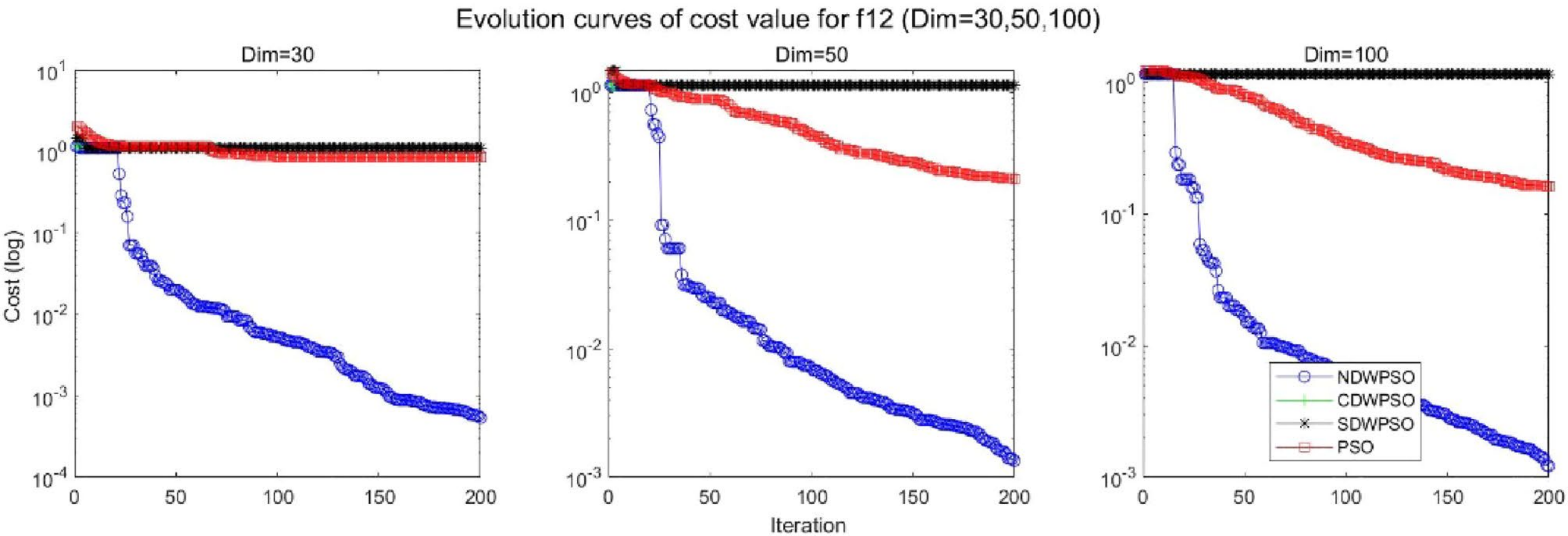
Evolution curve of NDWPSO and other PSO algorithms for f12 (Dim = 30,50,100).

**Figure 15:**
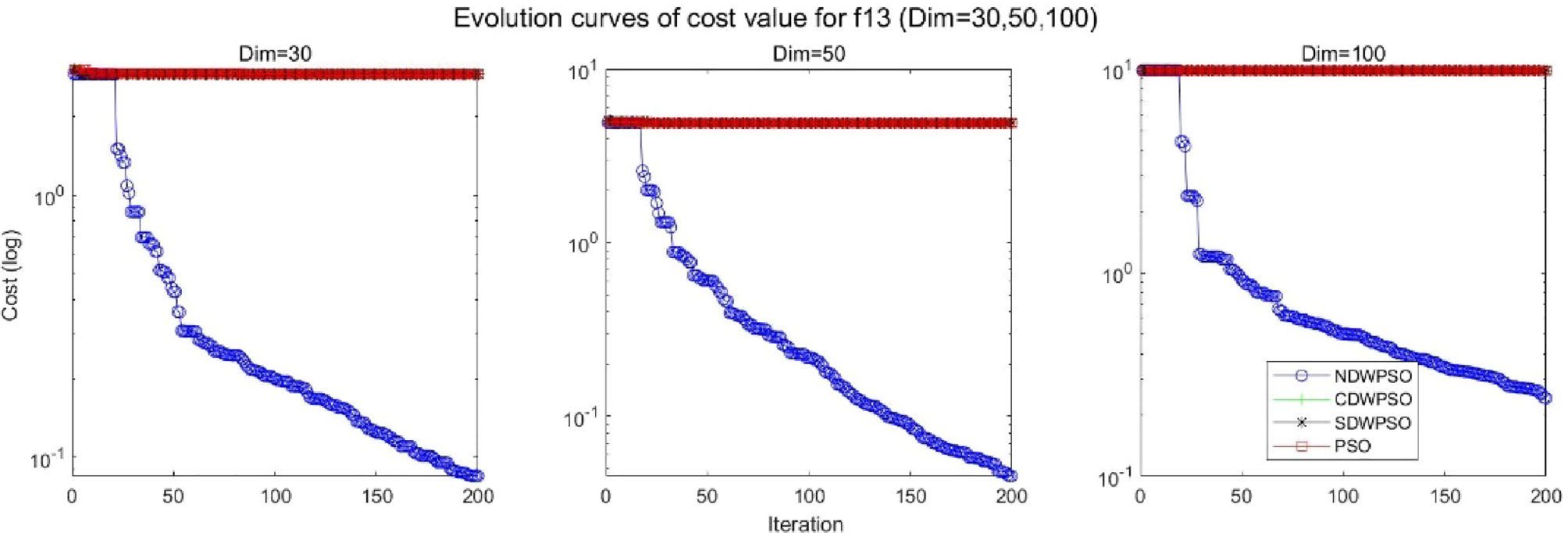
Evolution curve of NDWPSO and other PSO algorithms for f13 (Dim = 30,50,100).

**Figure 16:**
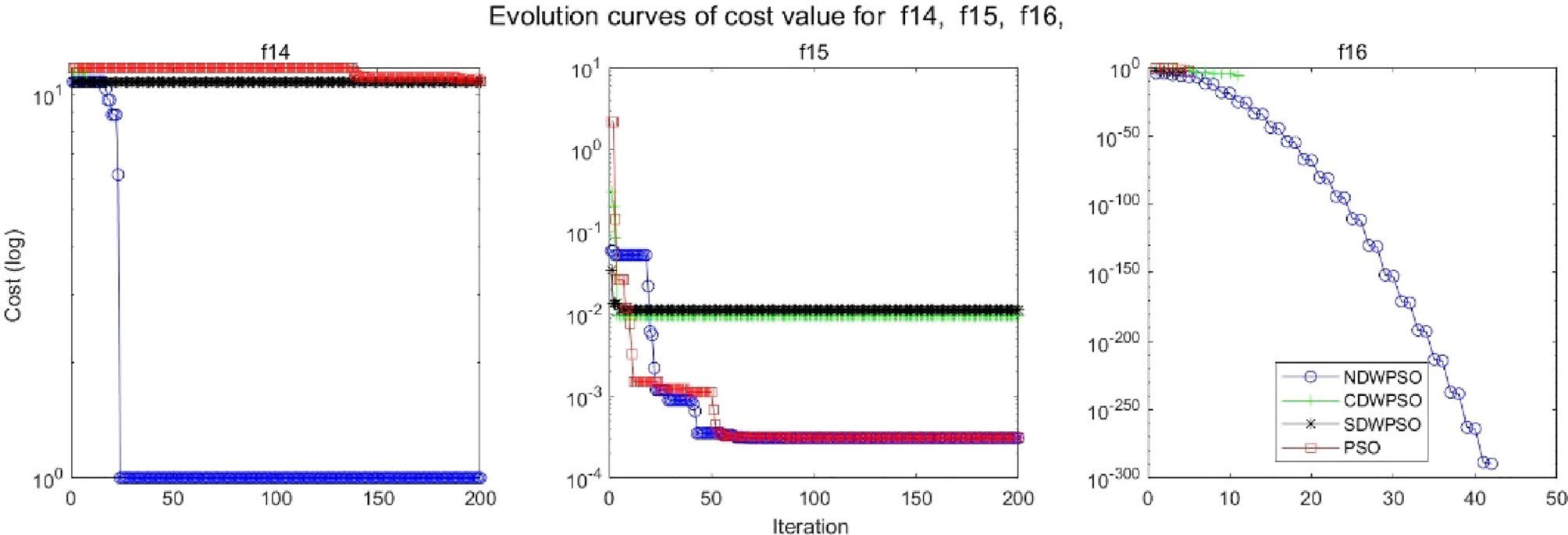
Evolution curve of NDWPSO and other PSO algorithms for f14, f15, f16.

**Figure 17:**
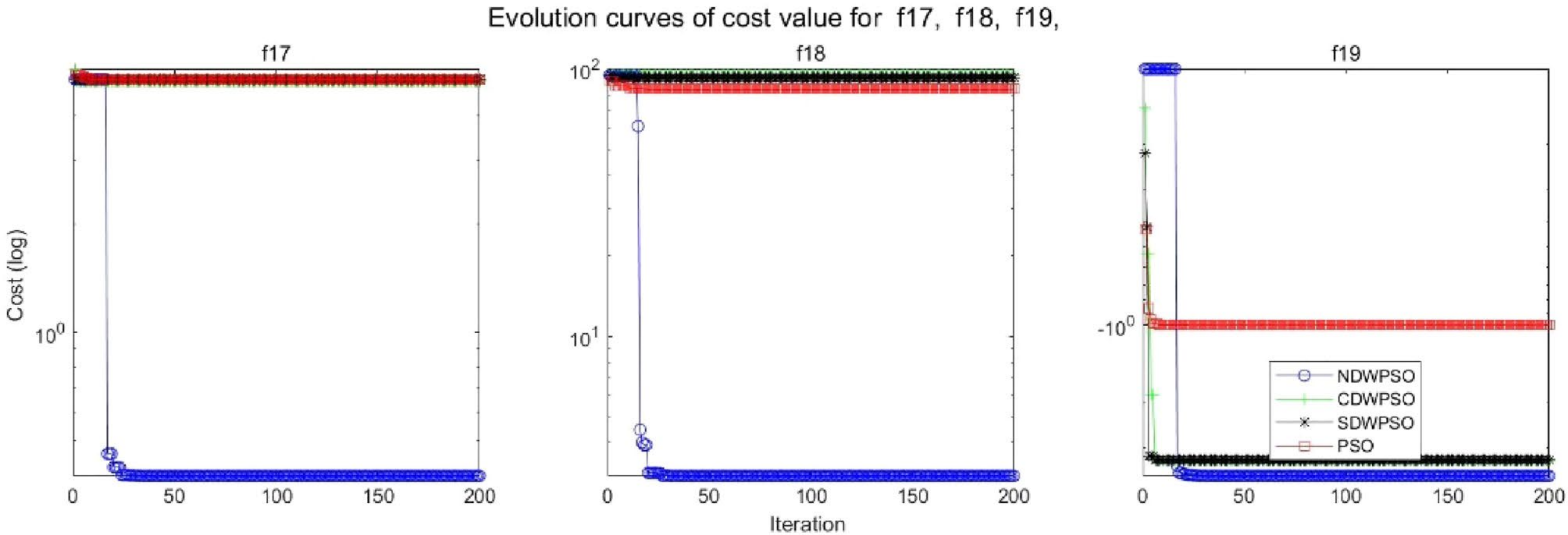
Evolution curve of NDWPSO and other PSO algorithms for f17, f18, f19.

**Figure 18:**
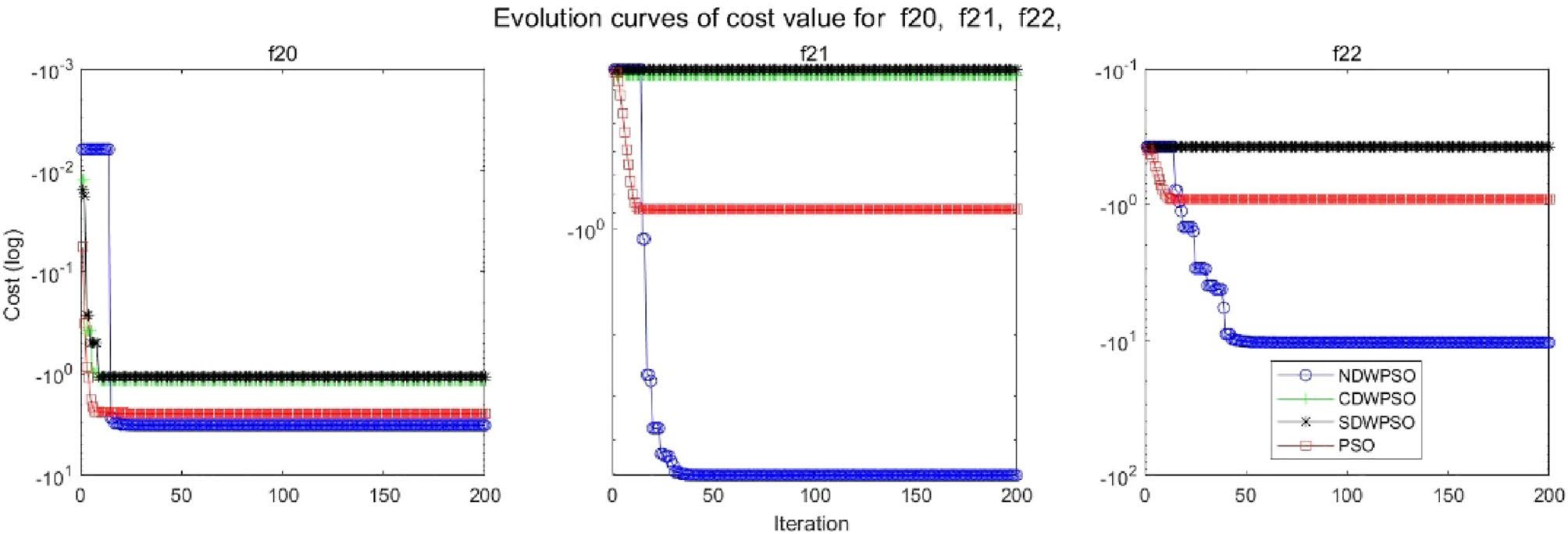
Evolution curve of NDWPSO and other PSO algorithms for f20, f21, f22.

**Figure 19:**
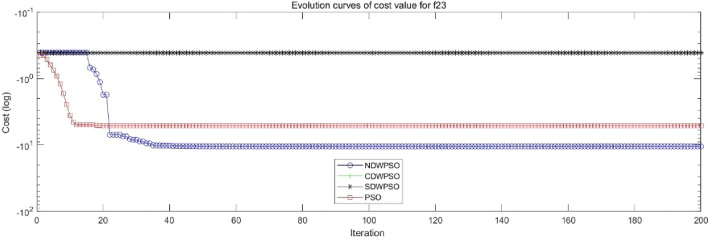
Evolution curve of NDWPSO and other PSO algorithms for f23.

In addition, the convergence curves of the 23 benchmark functions are shown in Figs. 3, 4, 5, 6, 7, 8, 9, 10, 11, 12, 13, 14, 15, 16, 17, 18 and 19. The NDWPSO algorithm has a faster convergence speed in the early stage of the search for processing functions f1-f6, f8-f14, f16, f17, and finds the global optimal solution with a smaller number of iterations. In the remaining benchmark function experiments, the NDWPSO algorithm shows no outstanding performance for convergence speed in the early iterations. There are two reasons of no outstanding performance in the early iterations. On one hand, the fixed-multimodal benchmark function has many disturbances and local optimal solutions in the whole search space. on the other hand, the initialization scheme based on elite opposition-based learning is still stochastic, which leads to the initial position far from the global optimal solution. The inertia weight based on chaotic mapping and the strategy of spiral updating can significantly improve the convergence speed and computational accuracy of the algorithm in the late search stage. Finally, the NDWPSO algorithm can find better solutions than other algorithms in the middle and late stages of the search.

To evaluate the performance of different PSO algorithms, a statistical test is conducted. Due to the stochastic nature of the meta-heuristics, it is not enough to compare algorithms based on only the mean and standard deviation values. The optimization results cannot be assumed to obey the normal distribution; thus, it is necessary to judge whether the results of the algorithms differ from each other in a statistically significant way. Here, the Wilcoxon non-parametric statistical test^45^ is used to obtain a parameter called *p*-value to verify whether two sets of solutions are different to a statistically significant extent or not. Generally, it is considered that *p* ≤ 0.5 can be considered as a statistically significant superiority of the results. The *p*-values calculated in Wilcoxon’s rank-sum test comparing NDWPSO and other PSO algorithms are listed in Table 3 for all benchmark functions. The *p*-values in Table 3 additionally present the superiority of the NDWPSO because all of the *p*-values are much smaller than 0.5.

**Table 3:** Results of the p-value for the Wilcoxon rank-sum test on benchmark functions.

	F1	F2	F3	F4	F5	F6	F7	F8
CDWPSO	4.25E−07	6.18E−07	5.17E−08	6.20E−08	7.09E−07	1.30E−07	0.029	3.94E−07
SDWPSO	8.40E−02	2.50E−01	3.41E−01	2.61E−01	7.09E−07	5.72E−07	2.79E−01	3.75E−07
PSO	8.49E−07	4.09E−07	3.68E−08	8.39E−08	6.26E−07	4.50E−07	3.58E−04	7.16E−07

	F9	F10	F11	F12	F13	F14	F15	F16
CDWPSO	0.041	0.195	0.030	3.61E−07	2.53E−07	8.53E−07	5.15E−04	2.94E−07
SDWPSO	6.66E−02	4.16E−01	2.00E−01	5.70E−07	3.74E−07	1.45E−07	2.74E−04	8.25E−07
PSO	1.50E−07	6.97E−07	4.57E−07	5.45E−07	1.34E−08	4.41E−08	1.15E−01	4.60E−01

	F17	F18	F19	F20	F21	F22	F23	
CDWPSO	4.57E−08	3.67E−07	3.62E−07	6.08E−07	6.05E−07	1.11E−07	2.83E−08	
SDWPSO	6.40E−07	4.75E−07	8.53E−07	5.78E−07	6.40E−07	9.60E−07	4.87E−07	
PSO	1.35E−01	5.94E−07	6.51E−09	6.58E−05	9.15E−08	6.10E−07	1.28E−06	

In general, the NDWPSO has the fastest convergence rate when finding the global optimum from Figs. 3, 4, 5, 6, 7, 8, 9, 10, 11, 12, 13, 14, 15, 16, 17, 18 and 19, and thus we can conclude that the NDWPSO is superior to the other PSO variants during the process of optimization.

### Comparison experiments between NDWPSO and other intelligent algorithms

**Figure 20:**
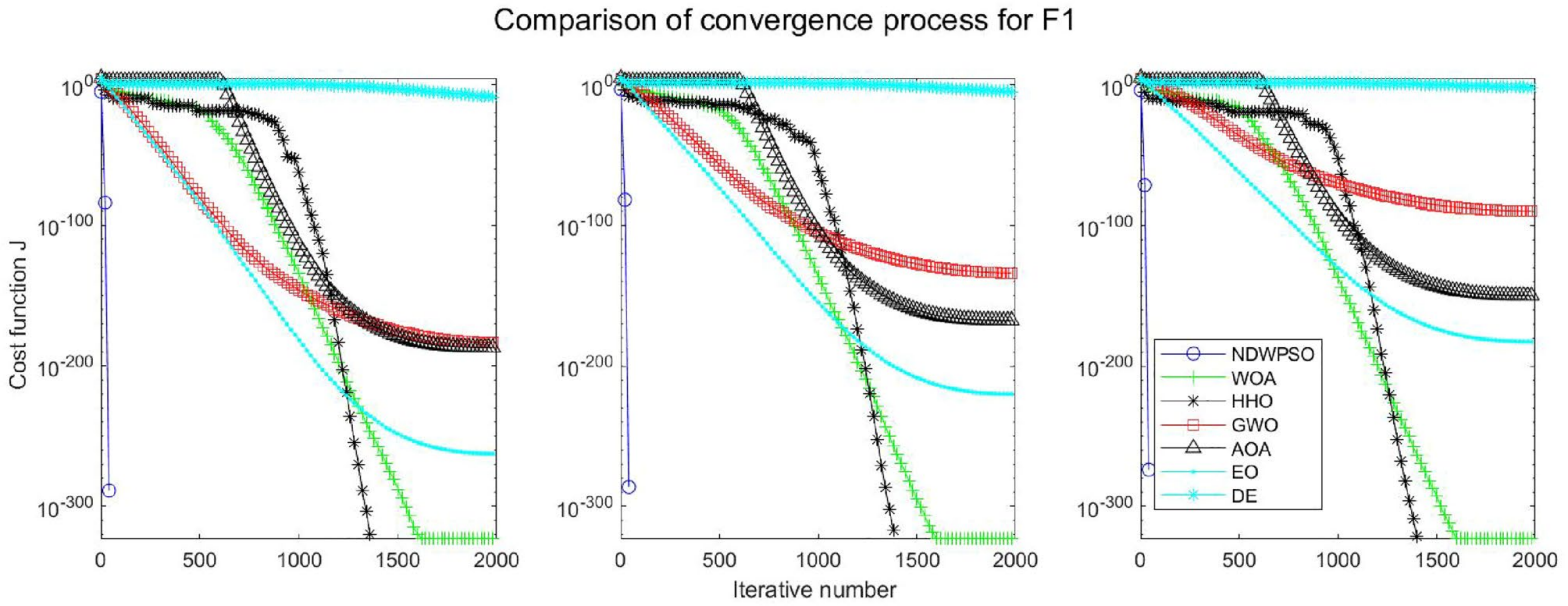
Evolution curve of NDWPSO and other algorithms for f1 (Dim = 30,50,100).

**Figure 21:**
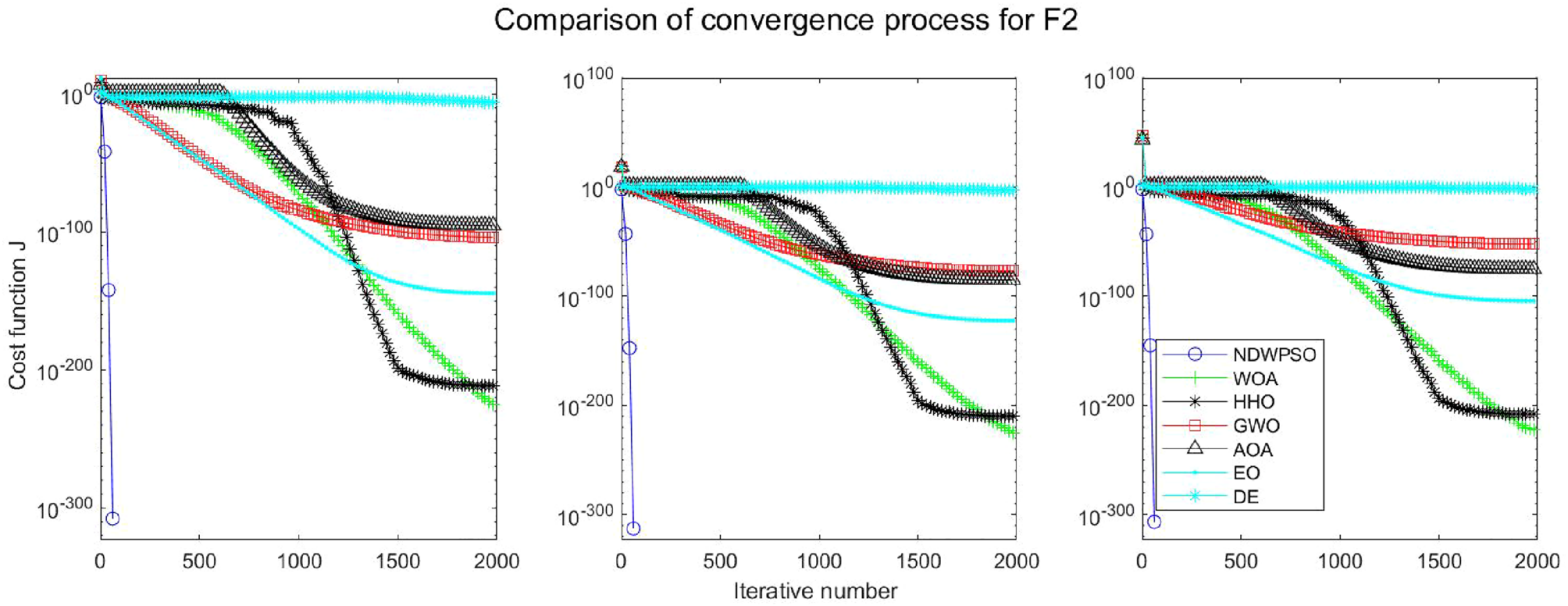
Evolution curve of NDWPSO and other algorithms for f2 (Dim = 30,50,100).

**Figure 22:**
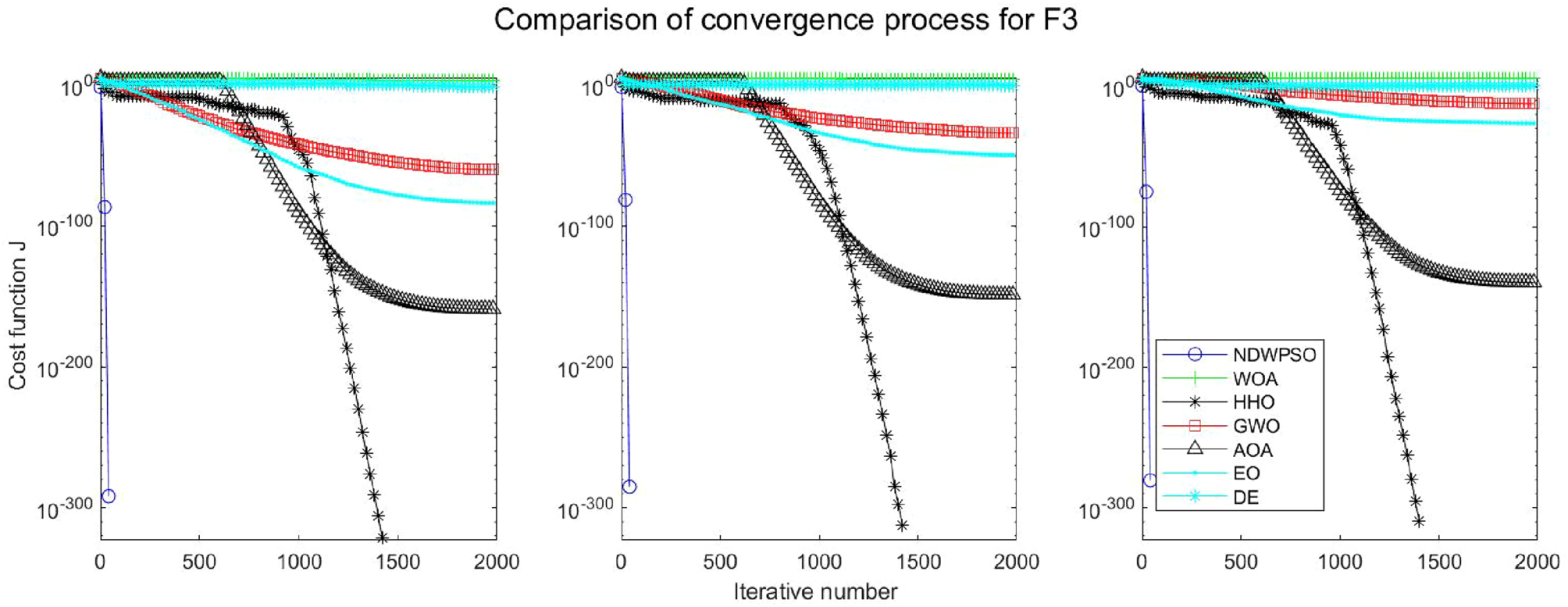
Evolution curve of NDWPSO and other algorithms for f3(Dim = 30,50,100).

**Figure 23:**
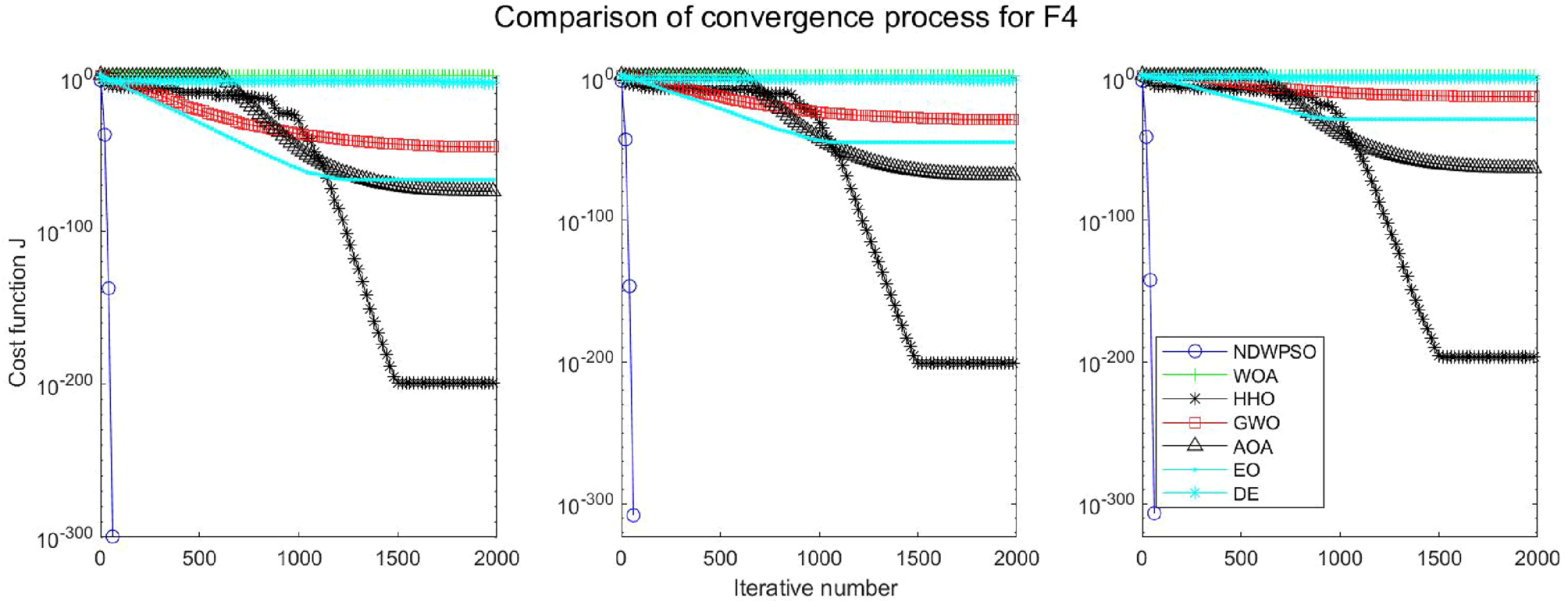
Evolution curve of NDWPSO and other algorithms for f4 (Dim = 30,50,100).

**Figure 24:**
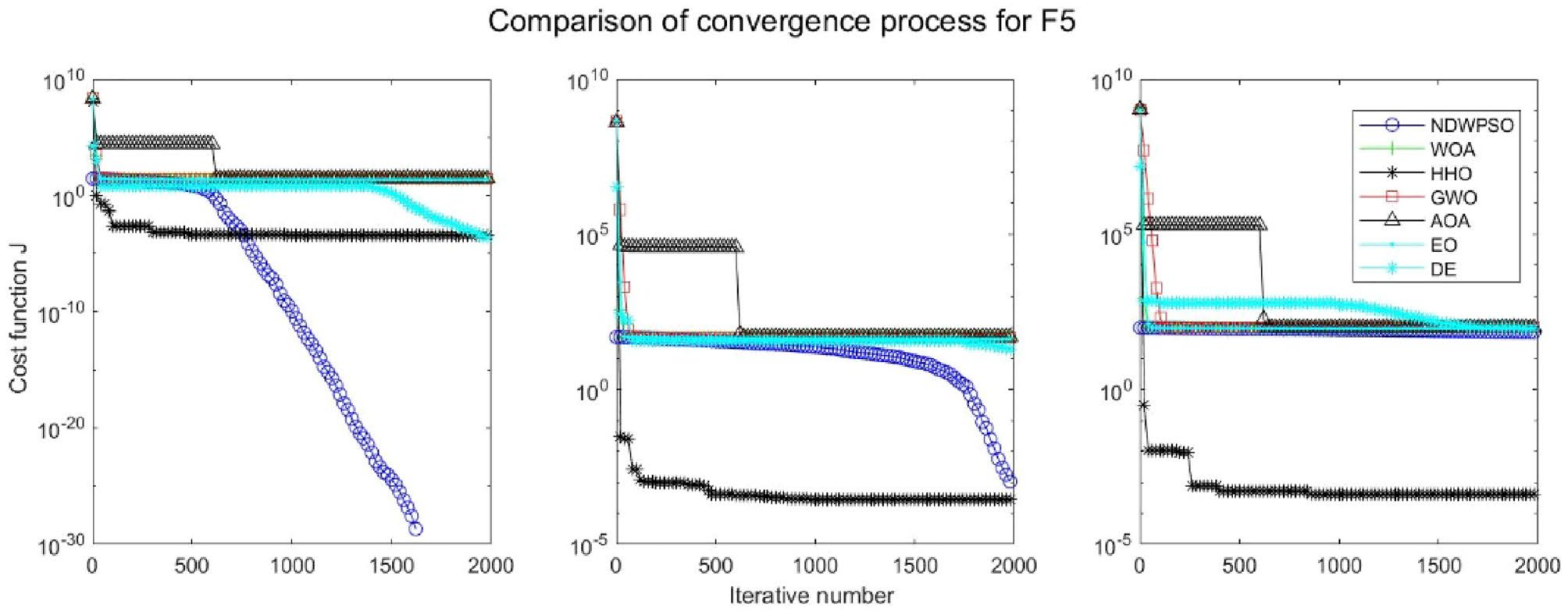
Evolution curve of NDWPSO and other algorithms for f5 (Dim = 30,50,100).

**Figure 25:**
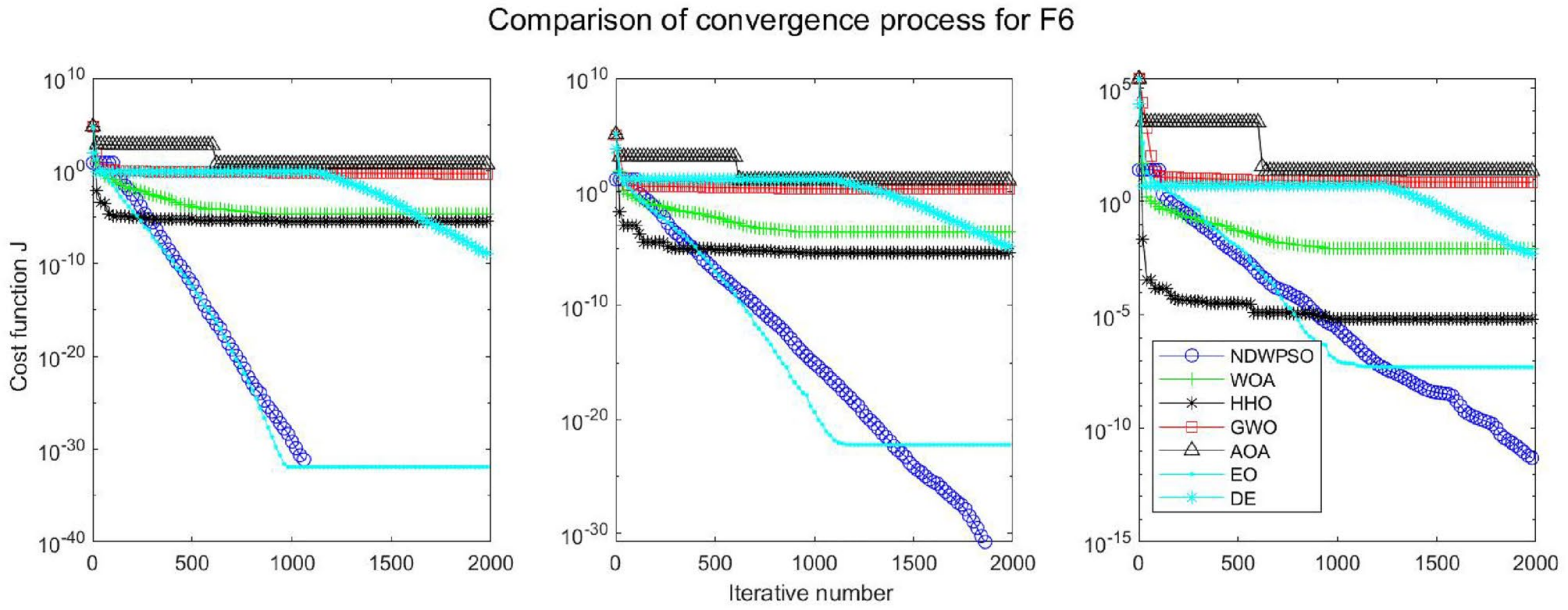
Evolution curve of NDWPSO and other algorithms for f6 (Dim = 30,50,100).

**Figure 26:**
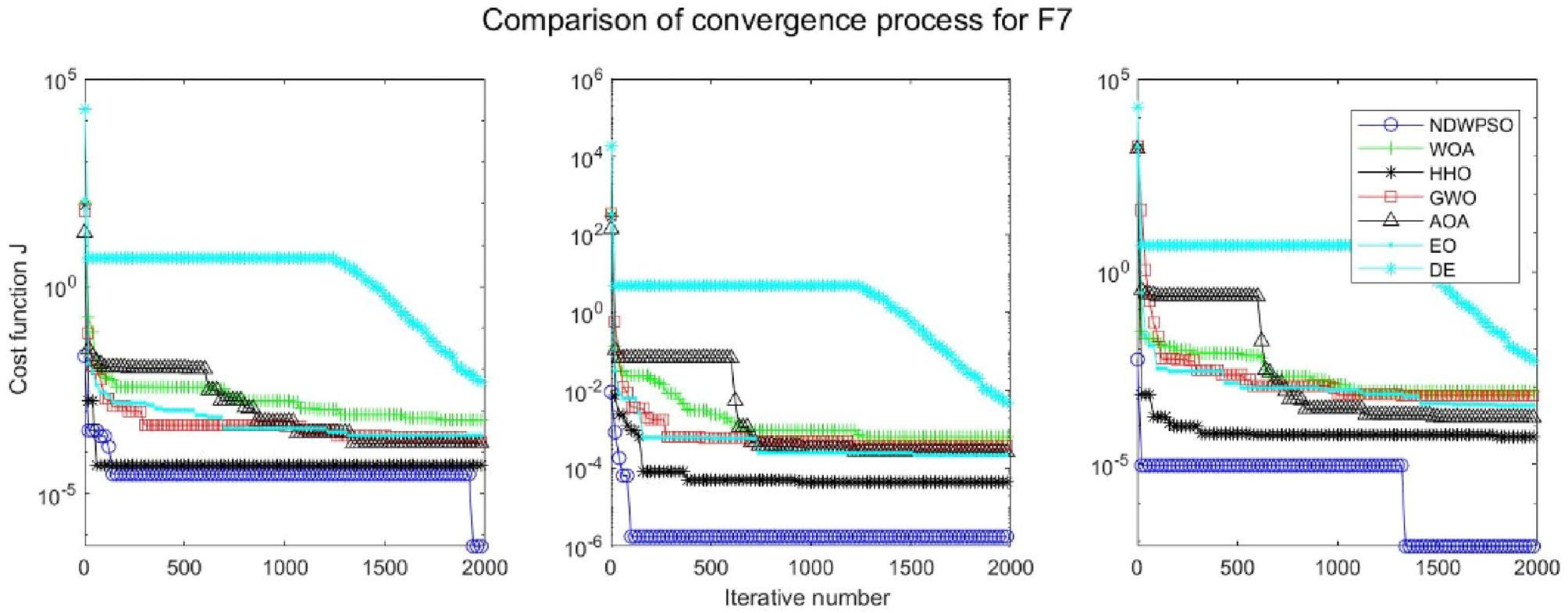
Evolution curve of NDWPSO and other algorithms for f7 (Dim = 30,50,100).

**Figure 27:**
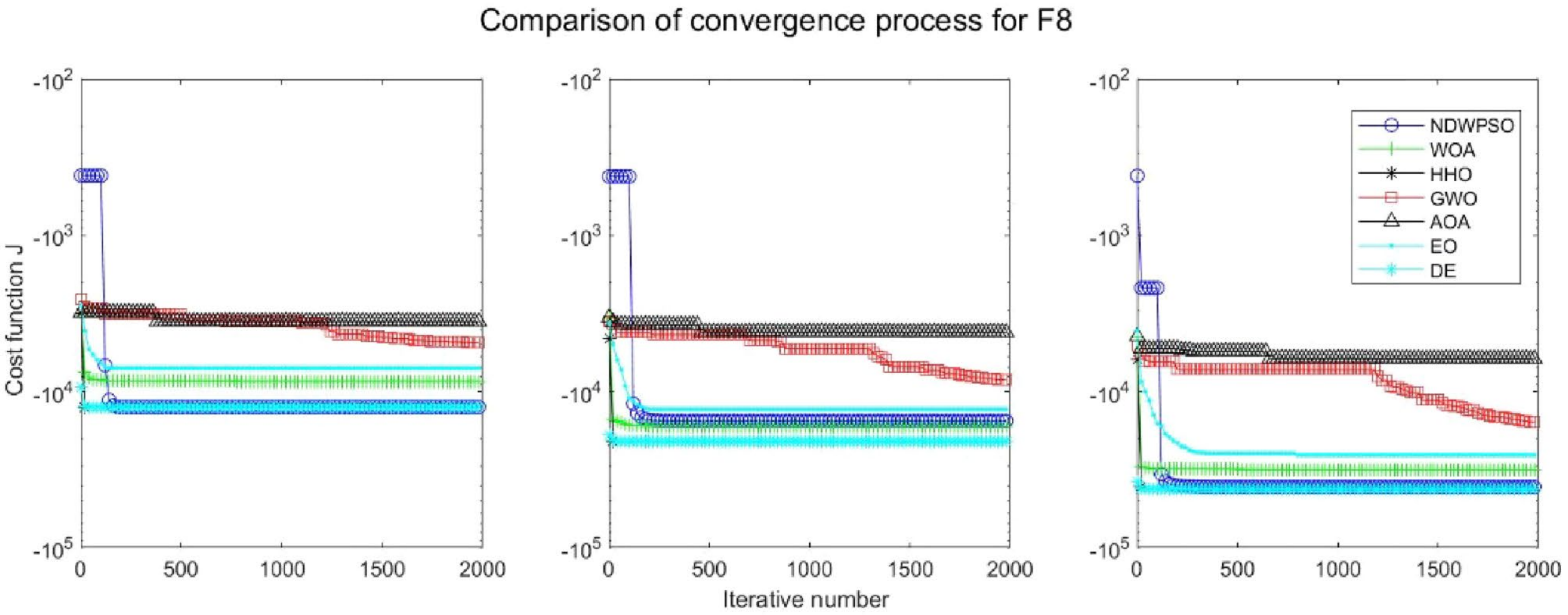
Evolution curve of NDWPSO and other algorithms for f8 (Dim = 30,50,100).

**Figure 28:**
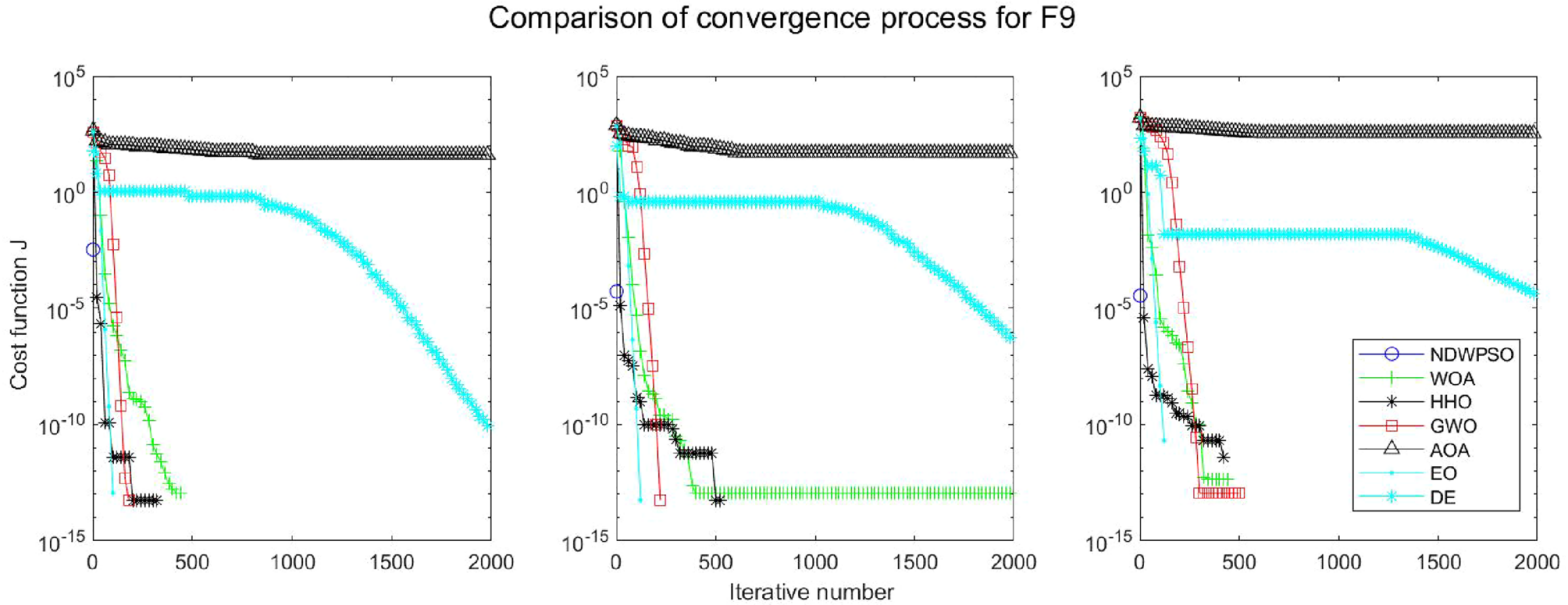
Evolution curve of NDWPSO and other algorithms for f9(Dim = 30,50,100).

**Figure 29:**
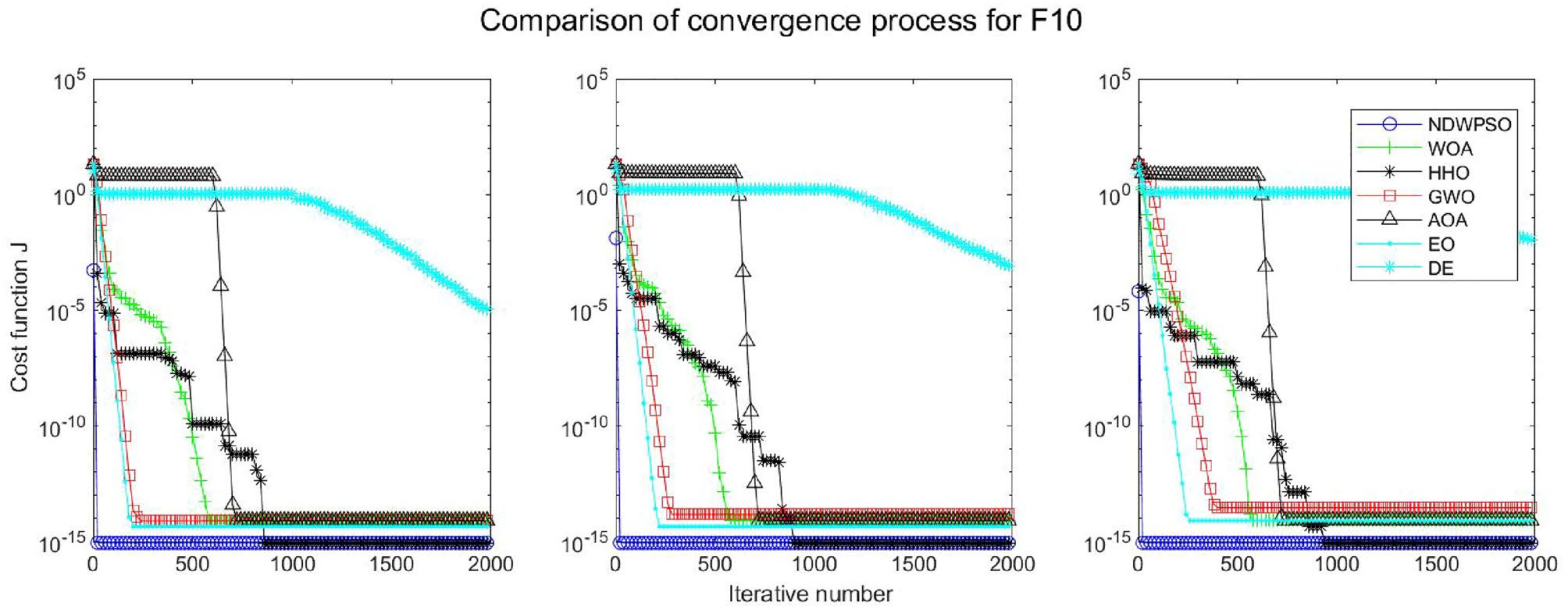
Evolution curve of NDWPSO and other algorithms for f10 (Dim = 30,50,100).

**Figure 30:**
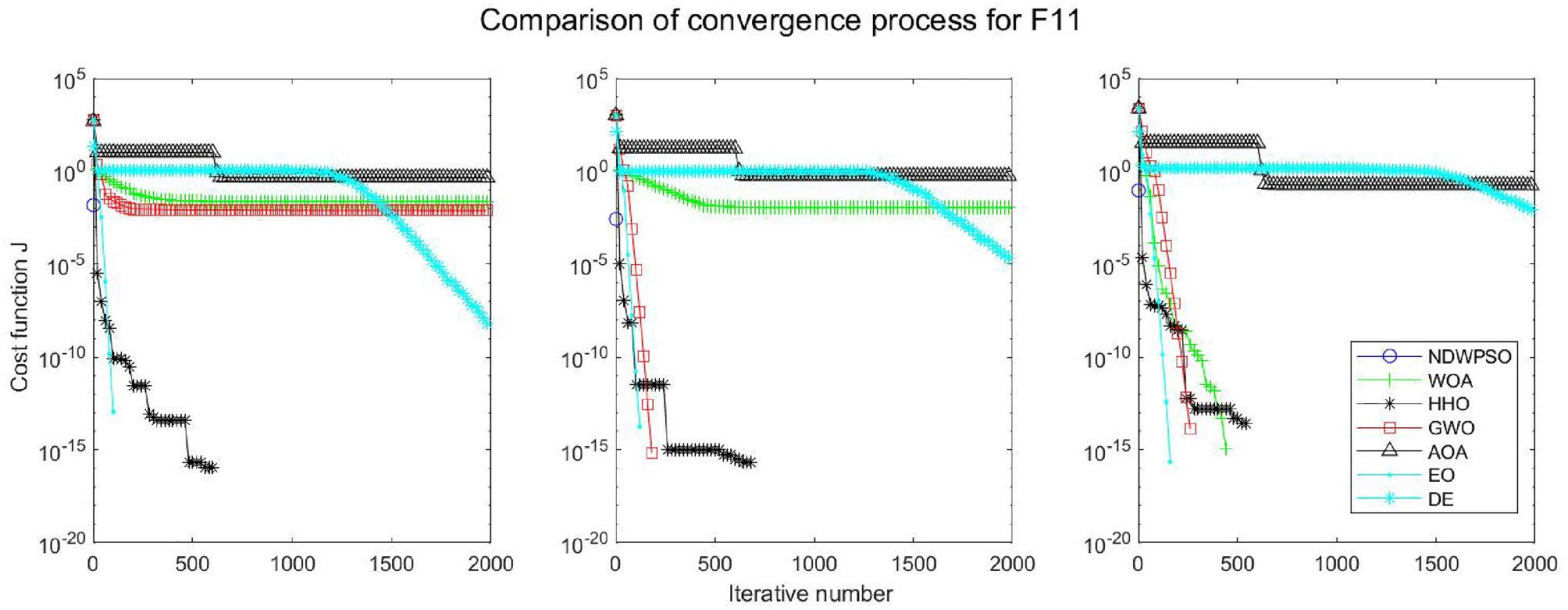
Evolution curve of NDWPSO and other algorithms for f11 (Dim = 30,50,100).

**Figure 31:**
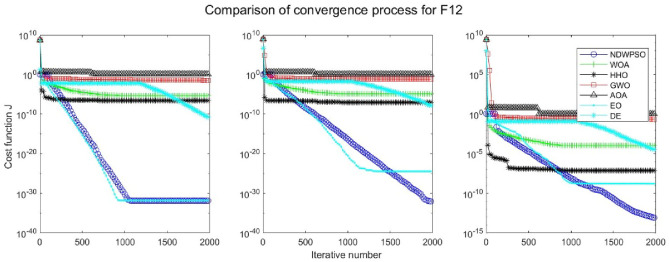
Evolution curve of NDWPSO and other algorithms for f12 (Dim = 30,50,100).

**Figure 32:**
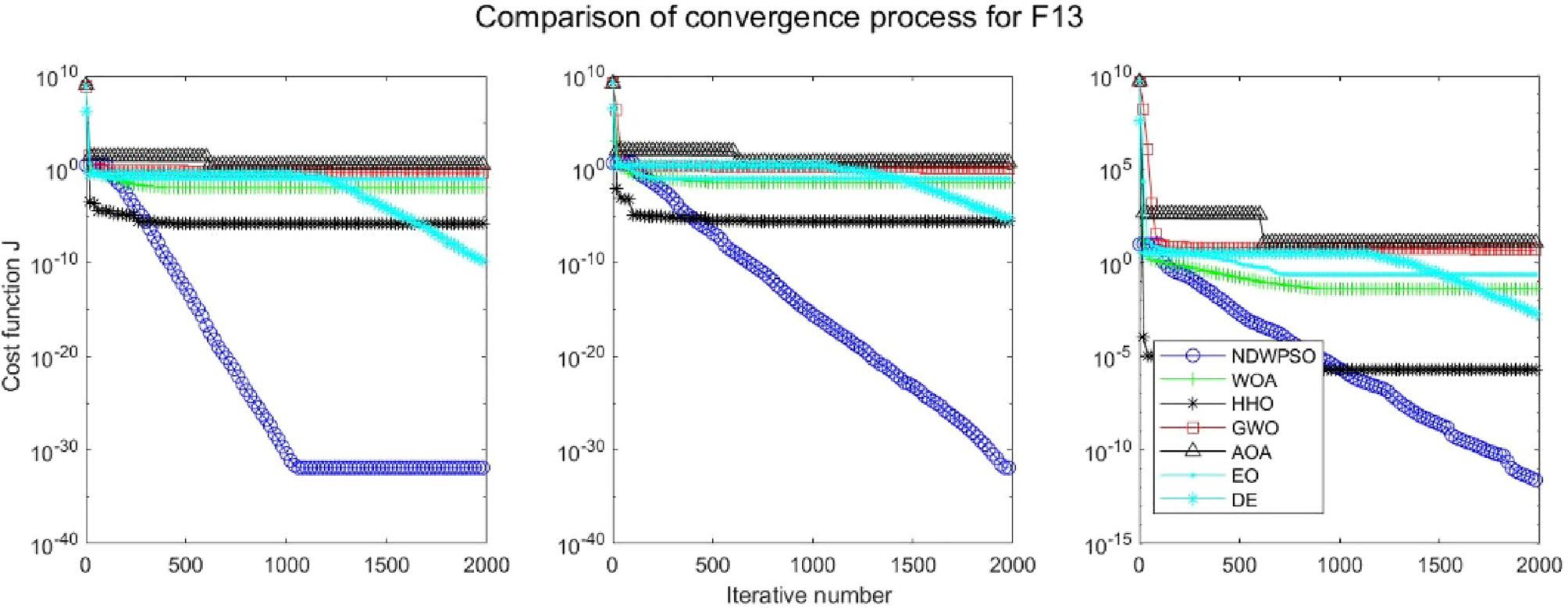
Evolution curve of NDWPSO and other algorithms for f13 (Dim = 30,50,100).

**Figure 33:**
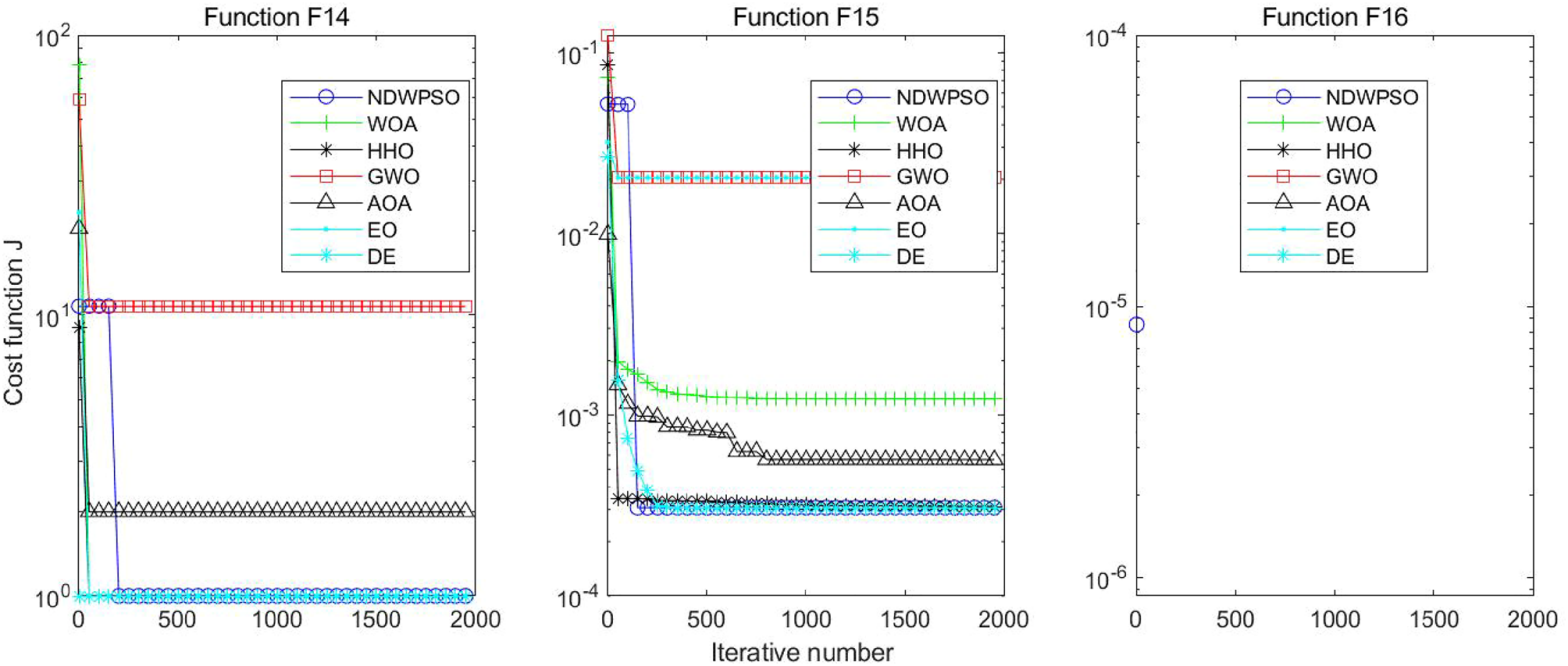
Evolution curve of NDWPSO and other algorithms for f14, f15, f16.

**Figure 34:**
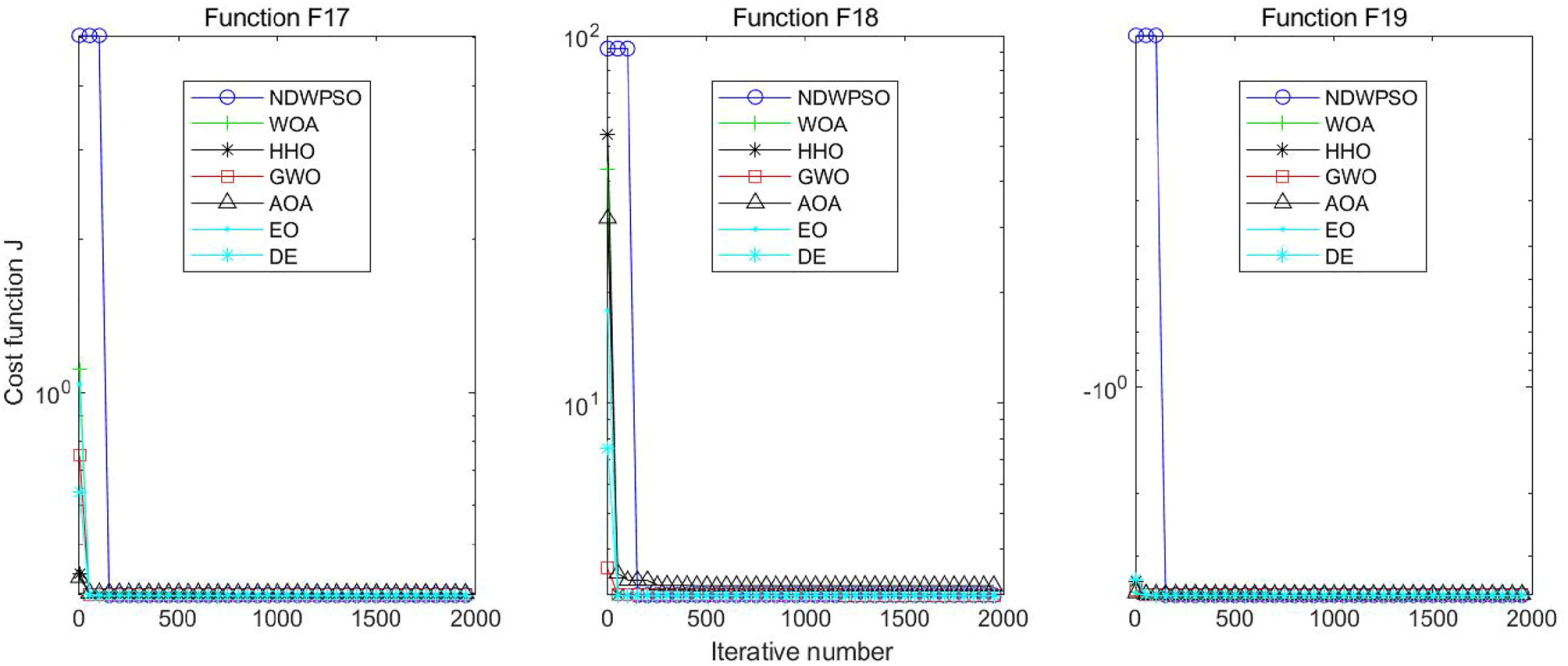
Evolution curve of NDWPSO and other algorithms for f17, f18, f19.

**Figure 35:**
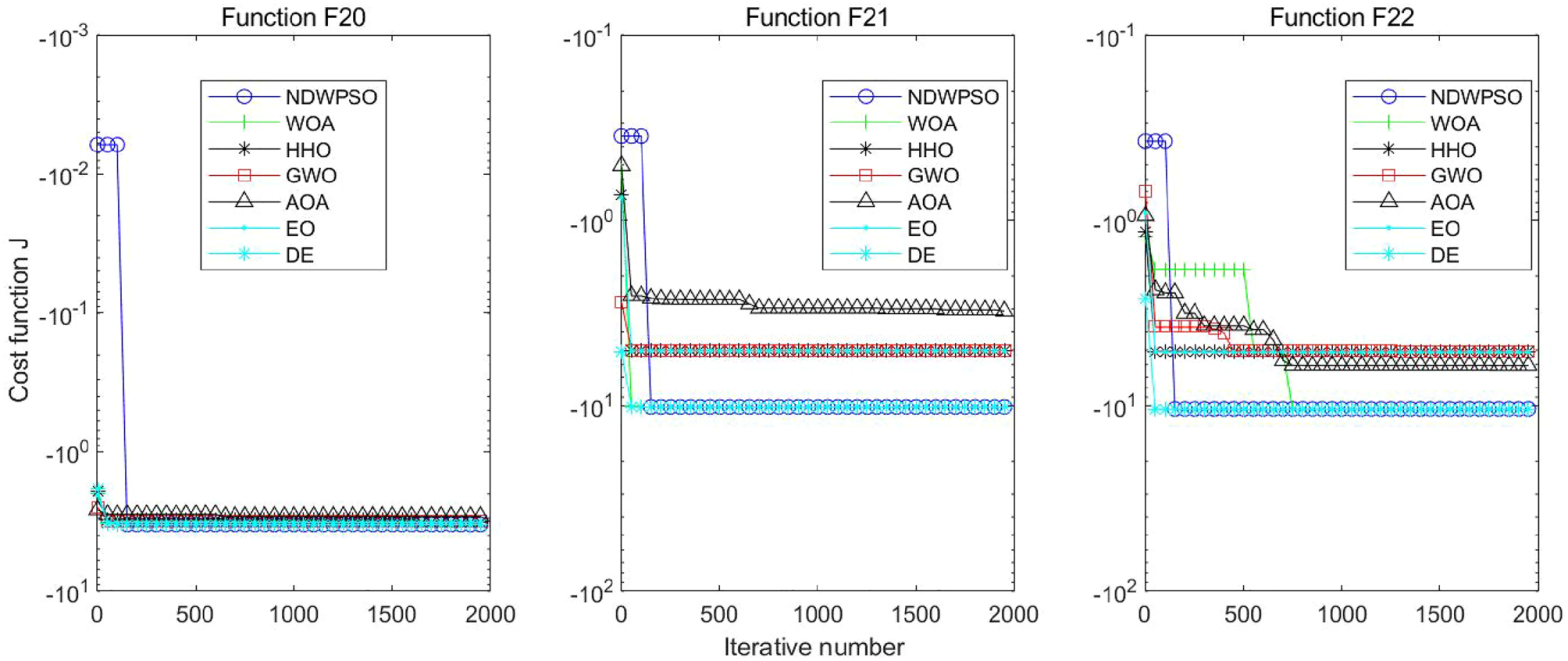
Evolution curve of NDWPSO and other algorithms for f20, f21, f22.

**Figure 36:**
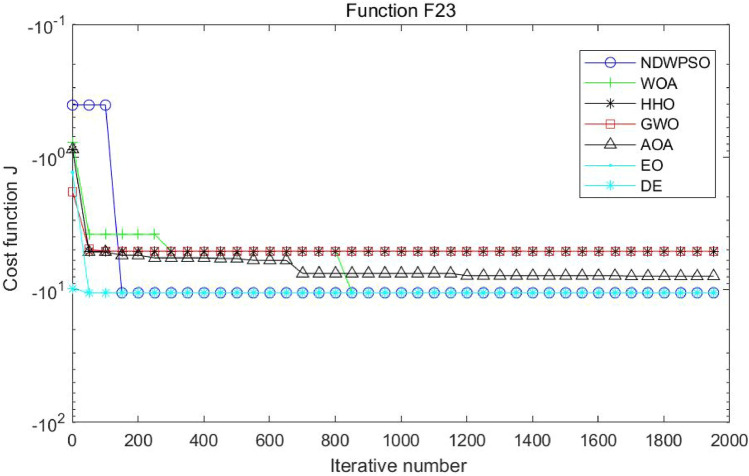
Evolution curve of NDWPSO and other algorithms for f23.

Experiments are conducted to compare NDWPSO with several other intelligent algorithms (WOA, HHO, GWO, AOA, EO and DE). The experimental object is 23 benchmark functions, and the experimental parameters of the NDWPSO algorithm are set the same as in Experiment 4.1. The maximum number of iterations of the experiment is increased to 2000 to fully demonstrate the performance of each algorithm. Each algorithm is repeated 30 times individually. The parameters of the relevant intelligent algorithms in the experiments are set as shown in Table 4. To ensure the fairness of the algorithm comparison, all parameters are concerning the original parameters in the relevant algorithm literature. The experimental results are shown in Tables 5, 6, 7 and 8 and Figs. 20, 21, 22, 23, 24, 25, 26, 27, 28, 29, 30, 31, 32, 33, 34, 35 and 36.

**Table 4:** Parameter settings for algorithms.

Algorithms	Parameter
WOA	$r1$ and $r2$ are random numbers in the range [0,1] $a$ variable decreases linearly from 2 to 0 $a2$ linearly decreases from -1 to -2
HHO	${E}_{0}$ variable changes from -1 to 1 (Default)
GWO	$r1$ and $r2$ are random numbers in the range [0,1] $a$ variable decreases linearly from 2 to 0
AOA	$C1=2,C2=6,$ $C3=1,C4=2,$(CEC and engineering problems) $u=0.9,l=0.1,$ $TF=0.5,p=0.5$
EO	${\alpha }_{1}=2, {\alpha }_{2}=1, GP=0.5;$
DE	F = 0.9, CR = 0.4

**Table 5:** Optimization results and comparison for functions(f_1_-f_13_) with Dim = 30.

Fun	Criteria	WOA	HHO	GWO	AOA	EO	DE	NDWPSO
f_1_	Ave	1.5e−323	**0**	1.1e−134	5.3e−166	5.2e−194	7.5E−09	**0**
	S.D	0	0	2.0e−134	0	0	8.0E−09	0
f_2_	Ave	6.2e−216	1.1e−196	1.32e−77	2.92e−94	7.9e−109	1.6E−05	**0**
	S.D	0	0	2.27e−77	1.53e−93	1.7e−108	8.8E−06	0
f_3_	Ave	3.80e + 03	3.05e−312	3.01e−38	2.4e−146	1.38e−51	3.9E−01	**0**
	S.D	3.28e + 03	0	9.36e−38	1.0e−145	4.30e−51	3.7E−01	0
f_4_	Ave	1.60e + 01	6.09e−184	3.33e−33	1.75e−75	1.85e−47	9.9E−03	**0**
	S.D	1.76e + 01	0	5.45e−33	9.21e−75	5.74e−47	7.3E−03	0
f_5_	Ave	2.59e + 01	**4.85e−04**	2.62e + 01	2.89e + 01	2.31e + 01	1.5E + 01	1.45e + 01
	S.D	0.25	6.71e−04	0.76	5.13e−02	0.17	8.1E + 00	1.72
f_6_	Ave	6.62e−04	5.97e−06	0.44	4.94	**2.26e−23**	6.3E−09	1.33e−17
	S.D	3.63e−04	9.46e−06	0.27	0.54	5.21e−23	5.8E−09	4.37e−17
f_7_	Ave	0.49	0.60	0.43	0.49	0.53	5.1E−02	**0.42**
	S.D	0.27	0.24	0.28	0.28	0.25	5.9E−02	0.26
f_8_	Ave	−1.21E + 04	**−1.26E + 04**	−5.90E + 03	−3.82E + 03	−9.01E + 03	−1.3E + 04	−7.02E + 03
	S.D	7.82e + 02	2.96e−02	9.05e + 02	4.69e + 02	7.13e + 02	6.3E−10	1.69e + 03
f_9_	Ave	**0**	**0**	0.30	4.12e + 01	**0**	3.2E−10	**0**
	S.D	0	0	1.64	4.09e + 01	0	2.7E−10	0
f_10_	Ave	3.61e−15	**8.88e−16**	8.82e−15	4.79e−15	4.44e−15	2.4E−05	**8.88e−16**
	S.D	2.54e−15	0	1.99e−15	1.07e−15	0	1.6E−05	0
f_11_	Ave	6.94e−04	**0**	**0**	3.97e−02	**0**	2.1E−07	**0**
	S.D	3.74e−03	0	0	0.13	0	5.7E−07	0
f_12_	Ave	1.04e−03	2.38e−07	2.74e−02	0.70	**4.22e−24**	1.2E−10	1.42e−17
	S.D	3.49e−03	2.72e−07	1.29e−02	0.18	1.25e−23	1.7E−10	3.75e−17
f_13_	Ave	1.48e−02	3.42e−06	0.33	2.72	9.79e−03	2.4E−09	**7.50e−15**
	S.D	2.63e−02	3.77e−06	0.19	0.34	2.73e−02	2.5E−09	2.33e−14

Significant values in bold.

**Table 6:** Optimization results and comparison for functions for(f_1_-f_13_) Dim = 50.

Fun	Criteria	WOA	HHO	GWO	AOA	EO	DE	NDWPSO
f_1_	Ave	**0**	**0**	3.4e−117	6.9e−159	1.2e−192	5.8E−05	**0**
	S.D	0	0	9.4e−117	2.5e−158	0	2.8E−05	0
f_2_	Ave	8.0e−223	2.35e−202	2.55e−68	2.02e−86	6.4e−109	1.7E−03	**0**
	S.D	0	0	2.64e−68	1.06e−85	9.9e−109	7.4E−04	0
f_3_	Ave	2.46e + 04	5.36e−299	3.15e−26	3.3e−143	1.56e−40	1.8E + 01	**0**
	S.D	1.46e + 04	0	1.61e−25	1.7e−142	8.08e−40	1.6E + 01	0
f_4_	Ave	3.43e + 01	1.34e−192	2.81e−26	1.13e−68	2.49e−39	2.1E−01	**0**
	S.D	3.26e + 01	0	5.35e−26	6.06e−68	1.23e−38	1.0E−01	0
f_5_	Ave	4.56e + 01	**3.09e−04**	4.65e + 01	4.88e + 01	4.25e + 01	4.2E + 01	3.07e + 01
	S.D	0.21	3.78e−04	0.77	7.85e−01	0.26	8.0E + 00	2.03
f_6_	Ave	1.26e−03	2.95e−06	1.29	9.09	3.74e−15	4.0E−05	**6.77e−16**
	S.D	5.48e−04	3.72e−06	0.43	0.57	1.17e−15	2.3E−05	1.61e−15
f_7_	Ave	0.64	0.49	0.45	0.51	0.62	5.1E−02	**0.43**
	S.D	0.26	0.29	0.28	0.26	0.26	5.9E−02	0.28
f_8_	Ave	−2.07E + 04	**−2.09E + 04**	−9.42E + 03	−5.02E + 03	−1.51E + 04	−2.10E + 04	−1.03E + 04
	S.D	3.20e + 02	6.40e−03	1.23e + 03	4.52e + 02	9.73e + 02	1.1E−05	2.57e + 03
f_9_	Ave	3.79e−15	**0**	**0**	2.27e + 01	**0**	8.3E−05	**0**
	S.D	2.04e−14	0	0	4.72e + 01	0	2.0E−04	0
f_10_	Ave	3.85e−15	**8.88e−16**	1.36e−14	5.74e−15	4.44e−15	1.5E−03	**8.88e−16**
	S.D	2.26e−15	0	2.54e−15	1.71e−15	0	4.0E−04	0
f_11_	Ave	6.15e−04	**0**	1.09e−03	1.83e−02	**0**	4.6E−05	**0**
	S.D	3.31e−03	0	5.86e−03	9.83e−02	0	2.1E−05	0
f_12_	Ave	6.93e−05	5.68e−08	4.83e−02	0.80	2.07e−03	6.4E−07	**5.72e−15**
	S.D	1.75e−05	6.98e−08	1.93e−02	0.12	1.12e−02	6.1E−07	1.91e−14
f_13_	Ave	1.30e−02	1.40e−06	0.97	4.96	3.06e−02	1.5E−05	**6.03e−14**
	S.D	1.88e−02	2.01e−06	0.26	0.30	5.85e−02	2.0E−05	5.80e−14

Significant values in bold.

**Table 7:** Optimization results and comparison for functions for(f_1_-f_13_) Dim = 100.

Fun	Criteria	WOA	HHO	GWO	AOA	EO	DE	NDWPSO
f_1_	Ave	**0**	**0**	4.99e−91	1.0e−153	2.9e−185	6.5E−02	**0**
	S.D	0	0	8.04e−91	5.7e−153	0	3.9E−02	0
f_2_	Ave	1.7e−227	1.6e−205	1.83e−53	2.93e−77	1.2e−105	4.1E−02	**0**
	S.D	0	0	1.57e−53	1.10e−76	1.2e−105	2.2E−02	0
f_3_	Ave	2.27e + 05	**0**	5.58e−14	2.2e−140	1.96e−24	1.4E + 02	**0**
	S.D	5.33e + 04	0	2.07e−13	1.1e−139	8.93e−24	1.2E + 02	0
f_4_	Ave	5.75e + 01	2.4e−199	4.19e−16	3.27e−65	2.12e−31	1.2E + 00	**0**
	S.D	2.90e + 01	0	1.16e−15	1.06e−64	4.41e−31	6.2E−01	0
f_5_	Ave	9.52e + 01	**1.89e−04**	9.66e + 01	9.88e + 01	9.15e + 01	9.8E + 01	7.83e + 01
	S.D	0.28	2.93e−04	0.95	4.81e−02	0.26	6.8E−01	2.08
f_6_	Ave	5.46e−03	1.03e−06	5.58	2.02e + 01	4.05e−09	5.1E−02	**7.35e−11**
	S.D	7.21e−04	7.84e−07	0.87	0.78	2.25e−09	5.9E−02	7.15e−11
f_7_	Ave	0.55	0.46	0.45	0.51	0.53	5.1E−02	**0.42**
	S.D	0.29	0.28	0.28	0.31	0.25	5.9E−02	0.30
f_8_	Ave	−4.13E + 04	**−4.19E + 04**	−1.68E + 04	−7.52E + 03	−2.98E + 04	−4.20E + 04	−2.27E + 04
	S.D	1.05e + 03	4.49e−03	2.067e + 03	9.45e + 03	1.96e + 03	3.3E−03	6.40e + 03
f_9_	Ave	**0**	**0**	0.29	3.79e−15	**0**	3.4E−03	**0**
	S.D	0	0	1.59	2.04e−14	0	7.5E−03	0
f_10_	Ave	4.67e−15	**8.88e−16**	2.42e−14	6.21e−15	5.38e−15	2.8E−02	**8.88e−16**
	S.D	2.42e−15	0	3.75e−15	1.78e−15	1.57e−15	1.1E−02	0
f_11_	Ave	1.46e−03	**0**	**0**	4.72e−03	**0**	3.5E−02	**0**
	S.D	7.88e−03	0	0	2.54e−02	0	2.0E−02	0
f_12_	Ave	7.26e−05	1.69–08	0.11	0.89	1.04e−03	4.5E−04	**1.12e−10**
	S.D	1.43e−05	4.60e−08	2.17e−02	9.00e−02	5.58e−03	3.5E−04	3.21e−10
f_13_	Ave	1.13e−02	4.07e−07	4.09	1.02e + 01	8.89e−02	4.6E−02	**1.74e−08**
	S.D	1.32e−02	5.58e−07	0.44	0.43	8.73e−02	7.6E−02	2.94e−08

Significant values in bold.

**Table 8:** Optimization results and comparison for function (f_14_–f_23_).

Fun	Criteria	WOA	HHO	GWO	AOA	EO	DE	NDWPSO
f_14_	Ave	1.654	**0.998**	5.044	1.162	**0.998**	**0.998**	**0.998**
	S.D	1.84	5.91e−11	4.27	0.44	0	0	1.25e−11
f_15_	Ave	5.69e−04	3.14e−04	1.67e−03	5.07e−04	2.37e−03	**3.07E−04**	**3.07e−04**
	S.D	3.32e−04	9.36e−06	4.50e−03	1.46e−04	6.01e−03	9.73E−20	1.90e−19
f_16_	Ave	**−1.0316**	**−1.0316**	**−1.0316**	**−1.0316**	**−1.0316**	**−1.0316**	**−1.0316**
	S.D	3.35e−13	2.86e−14	5.63e−10	1.35e−05	6.47e−16	0	6.66e−16
f_17_	Ave	**0.39789**	**0.39789**	0.3979	0.39793	**0.39789**	**0.39789**	**0.39789**
	S.D	0	8.78e−09	8.78e−09	4.98e−05	1.82e−04	0	0
f_18_	Ave	**3**	**3**	5.70	3.01	**3**	**3**	**3**
	S.D	1.31e−06	2.60e−10	1.45e + 01	1.66e−01	2.20e−15	9.82E−16	1.07e−15
f_19_	Ave	−3.8614	−3.8626	−3.8619	−3.8605	**−3.8628**	**−3.8628**	**−3.8628**
	S.D	2.56e−03	3.79e−04	2.38e−03	3.32e−03	2.64e−15	9.36E−16	2.67e−15
f_20_	Ave	−3.2345	−3.2273	−3.2623	−3.118	−3.2623	−3.3219	**−3.2784**
	S.D	7.67e−02	6.19e−02	6.66e−02	0.14	6.86e−02	5.92E−16	5.73e−02
f_21_	Ave	−9.13	−7.08	−9.12	−7.39	−8.11	−10.15	**−9.14**
	S.D	1.28	2.48	2.04	2.24	2.50	0	2.02
f_22_	Ave	−10.0028	−5.4408	**−10.0499**	−8.6794	−10.0031	−10.40	−9.3399
	S.D	1.51	1.32	1.32	1.98	1.51	1.87E−15	2.13
f_23_	Ave	−9.9949	−5.3082	**−10.5363**	−9.1959	−9.8153	−10.53	−9.4548
	S.D	1.62	0.97	1.93e−05	1.62	1.83	1.87E−15	2.16

Significant values in bold.

The experimental data of NDWPSO and other intelligent algorithms for handling 30, 50, and 100-dimensional benchmark functions (${f}_{1}-{f}_{13}$) are recorded in Tables 8, 9 and 10, respectively. The comparison data of fixed-multimodal benchmark tests (${f}_{14}-{f}_{23}$) are recorded in Table 11. According to the data in Tables 5, 6 and 7, the NDWPSO algorithm obtains 69.2%, 84.6%, and 84.6% of the best results for the benchmark function (${f}_{1}-{f}_{13}$) in the search space of three dimensions (Dim = 30, 50, 100), respectively. In Table 8, the NDWPSO algorithm obtains 80% of the optimal solutions in 10 fixed-multimodal benchmark functions.

**Table 9:** Comparison of results for welded beam design problem.

Algorithm	Optimal value for variables				Optimal cost	S.D
	${\text{h}}$	${\text{l}}$	${\text{t}}$	${\text{b}}$		
NDWPSO	2.06E−01	3.25E + 00	9.04E + 00	2.06E−01	**1.70E + 00**	**2.76E−09**
WOA	1.69E−01	5.23E + 00	8.48E + 00	2.34E−01	2.46E + 00	6.77E−01
HHO	1.87E−01	3.67E + 00	9.06E + 00	2.06E−01	2.01E + 00	2.15E−01
GWO	2.04E−01	3.30E + 00	9.04E + 00	2.06E−01	**1.70E + 00**	1.96E−03
AOA	3.07E−01	2.42E + 00	7.43E + 00	3.07E−01	3.14E + 00	4.70E−01
EO	2.06E−01	3.25E + 00	9.04E + 00	2.06E−01	**1.70E + 00**	4.80E−04

Significant values in bold.

**Table 10:** Comparison of results for pressure vessel design problem.

Algorithm	Optimal value for variables				Optimal cost	S.D
	${T}_{s}$	${T}_{h}$	${\text{R}}$	${\text{L}}$		
NDWPSO	7.78E−01	3.85E−01	4.03E + 01	2.00E + 02	**5.89E + 03**	**4.9732e−05**
WOA	1.20E + 00	6.64E−01	5.75E + 01	4.80E + 01	1.12E + 04	7.3624e + 03
HHO	1.15E + 00	5.44E−01	5.67E + 01	5.24E + 01	6.95E + 03	648.2539
GWO	7.79E−01	3.86E−01	4.03E + 01	2.00E + 02	6.08E + 03	352.7299
AOA	9.93E−01	5.07E−01	5.09E + 01	9.15E + 01	2.84E + 04	3.9074e + 04
EO	1.24E + 00	6.14E−01	6.44E + 01	1.37E + 01	6.65E + 03	5.0160e + 02

Significant values in bold.

**Table 11:** Comparison of results for the three-bar truss design problem.

Algorithm	Optimal value for variables		Optimal cost	S.D
	x_1_	x_2_		
NDWPSO	7.86E−01	4.07E−01	**2.63E + 02**	**1.36E−05**
WOA	8.10E−01	3.43E−01	2.64E + 02	1.46E + 00
HHO	7.92E−01	3.92E−01	2.64E + 02	3.61E−02
GWO	7.87E−01	4.12E−01	2.64E + 02	4.77E−03
AOA	7.92E−01	3.89E−01	2.64E + 02	4.32E−01
EO	7.88E−01	4.11E−01	2.64E + 02	1.25E−03

Significant values in bold.

The convergence curves of each algorithm are shown in Figs. 20, 21, 22, 23, 24, 25, 26, 27, 28, 29, 30, 31, 32, 33, 34, 35 and 36. The NDWPSO algorithm demonstrates two convergence behaviors when calculating the benchmark functions in 30, 50, and 100-dimensional search spaces. The first behavior is the fast convergence of NDWPSO with a small number of iterations at the beginning of the search. The reason is that the Iterative-mapping strategy and the position update scheme of dynamic weighting are used in the NDWPSO algorithm. This scheme can quickly target the region in the search space where the global optimum is located, and then precisely lock the optimal solution. When NDWPSO processes the functions ${f}_{1}-{f}_{4}$, and ${f}_{9}-{f}_{11}$, the behavior can be reflected in the convergence trend of their corresponding curves. The second behavior is that NDWPSO gradually improves the convergence accuracy and rapidly approaches the global optimal in the middle and late stages of the iteration. The NDWPSO algorithm fails to converge quickly in the early iterations, which is possible to prevent the swarm from falling into a local optimal. The behavior can be demonstrated by the convergence trend of the curves when NDWPSO handles the functions ${f}_{6}$, ${f}_{12}$, and ${f}_{13}$, and it also shows that the NDWPSO algorithm has an excellent ability of local search.

Combining the experimental data with the convergence curves, it is concluded that the NDWPSO algorithm has a faster convergence speed, so the effectiveness and global convergence of the NDWPSO algorithm are more outstanding than other intelligent algorithms.

### Experiments on classical engineering problems

Three constrained classical engineering design problems (welded beam design, pressure vessel design^43^, and three-bar truss design^38^) are used to evaluate the NDWPSO algorithm. The experiments are the NDWPSO algorithm and 5 other intelligent algorithms (WOA^36^, HHO, GWO, AOA, EO^41^). Each algorithm is provided with the maximum number of iterations and population size (${\text{Mk}}=500,\mathrm{ n}=40$), and then repeats 30 times, independently. The parameters of the algorithms are set the same as in Table 4. The experimental results of three engineering design problems are recorded in Tables 9, 10 and 11 in turn. The result data is the average value of the solved data.

#### Welded beam design

**Figure 37:**
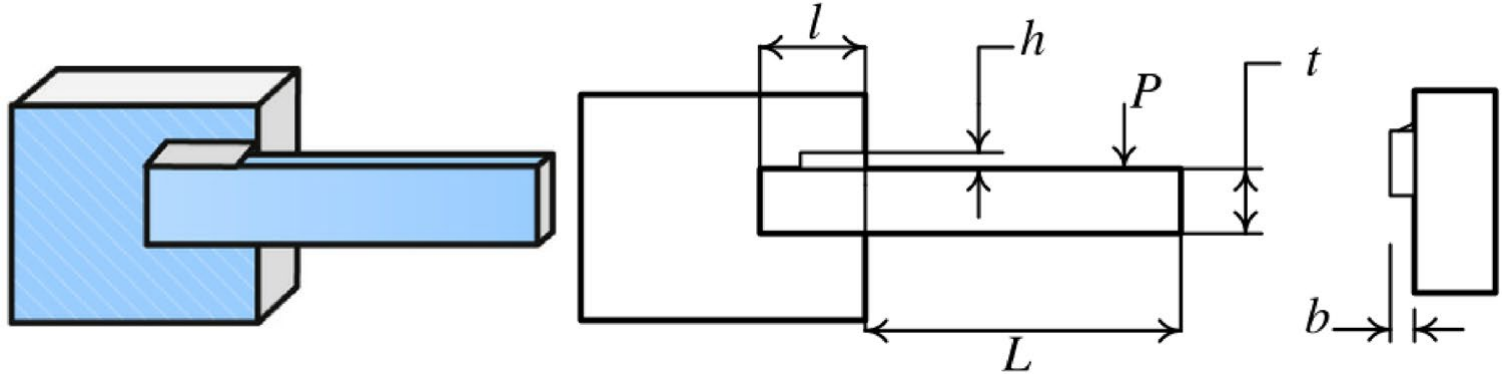
Welded beam design.

The target of the welded beam design problem is to find the optimal manufacturing cost for the welded beam with the constraints, as shown in Fig. 37. The constraints are the thickness of the weld seam (${\text{h}}$), the length of the clamped bar (${\text{l}}$), the height of the bar (${\text{t}}$) and the thickness of the bar (${\text{b}}$). The mathematical formulation of the optimization problem is given as follows:

$$\begin{aligned} & {\text{Consider}}\quad {\text{X}} = \left[ {x_{1} ,{ }x_{2} ,{ }x_{3} ,x_{4} \left] = \right[{\text{h}},{\text{ l}},{\text{ t}},{\text{ b}}} \right]; \\ & {\text{Objective function}}\quad {\text{f}}\left( {\text{x}} \right) = 1.10471x_{1}^{2} x_{2} + 0.04811x_{3} x_{4} \left( {14 + x_{2} } \right); \\ & {\text{Subject to}}\quad g_{1} \left( x \right) = \tau \left( x \right) - \tau_{max} \le 0; \\ & \quad \quad \quad \quad \quad g_{2} \left( x \right) = \sigma \left( x \right) - \sigma_{max} \le 0; \\ & \quad \quad \quad \quad \quad g_{3} \left( x \right) = \delta \left( x \right) - \delta_{max} \le 0; \\ & \quad \quad \quad \quad \quad g_{4} \left( x \right) = x_{1} - x_{4} \le 0; \\ & \quad \quad \quad \quad \quad g_{5} \left( x \right) = P - P_{c} \left( x \right) \le 0; \\ & \quad \quad \quad \quad \quad g_{6} \left( x \right) = 0.125 - x_{1} \le 0; \\ & \quad \quad \quad \quad \quad g_{7} \left( x \right) = 1.10471x_{1}^{2} + 0.004811x_{3} x_{4} \left( {14 + x_{2} } \right) - 5 \le 0; \\ & {\text{where}}\quad \quad \tau \left( x \right)_{ } = \sqrt {\tau^{^{\prime}2} + 2\tau^{\prime}\tau^{\prime\prime}\frac{{x_{2} }}{2R} + \tau^{2} } ,{ }\tau^{\prime}` = \frac{P}{{\sqrt 2 x_{1} x_{2} }} \\ & \quad \quad \quad \quad \tau^{\prime\prime} = \frac{MR}{J}, M = P\left( {L + \frac{{x_{2} }}{2}} \right) \\ & \quad \quad \quad \quad R_{ } = \sqrt {\frac{{x_{2}^{2} }}{4} + \left( {\frac{{x_{1} + x_{3} }}{2}} \right)^{2} } \\ & \quad \quad \quad \quad J = 2\left\{ {\sqrt 2 x_{1} x_{2} \left[ {\frac{{x_{2}^{2} }}{12} + \left( {\frac{{x_{1} + x_{3} }}{2}} \right)^{2} } \right]} \right\} \\ & \quad \quad \quad \quad \sigma \left( x \right)_{ } = \frac{6PL}{{x_{4} x_{3}^{2} }} \\ & \quad \quad \quad \quad \delta \left( x \right)_{ } = \frac{{4PL^{3} }}{{Ex_{3}^{3} x_{4} }} \\ & \quad \quad \quad \quad P_{c} \left( x \right) = \frac{{4.013E\sqrt {\frac{{x_{3}^{2} x_{4}^{6} }}{36}} }}{{L^{2} }}\left( {1 - \frac{{x_{3} }}{2L}\sqrt{\frac{E}{4G}} } \right) \\ & {\text{Variable range}}:\;0.1 \le x_{1} \le 2 \\ & \quad \quad \quad \quad \quad \quad \;0.1 \le x_{2} \le 10 \\ & \quad \quad \quad \quad \quad \quad \;0.1 \le x_{3} \le 10 \\ & \quad \quad \quad \quad \quad \quad \;0.1 \le x_{4} \le 2 \\ & \quad \quad \quad \quad \quad \quad \;P = 6000lb, L = 14in, E = 30 \times 10^{6} psi, G = 12 \times 10^{6} psi \\ \end{aligned}$$

In Table 9, the NDWPSO, GWO, and EO algorithms obtain the best optimal cost. Besides, the standard deviation (SD) of t NDWPSO is the lowest, which means it has very good results in solving the welded beam design problem.

#### Pressure vessel design

**Figure 38:**
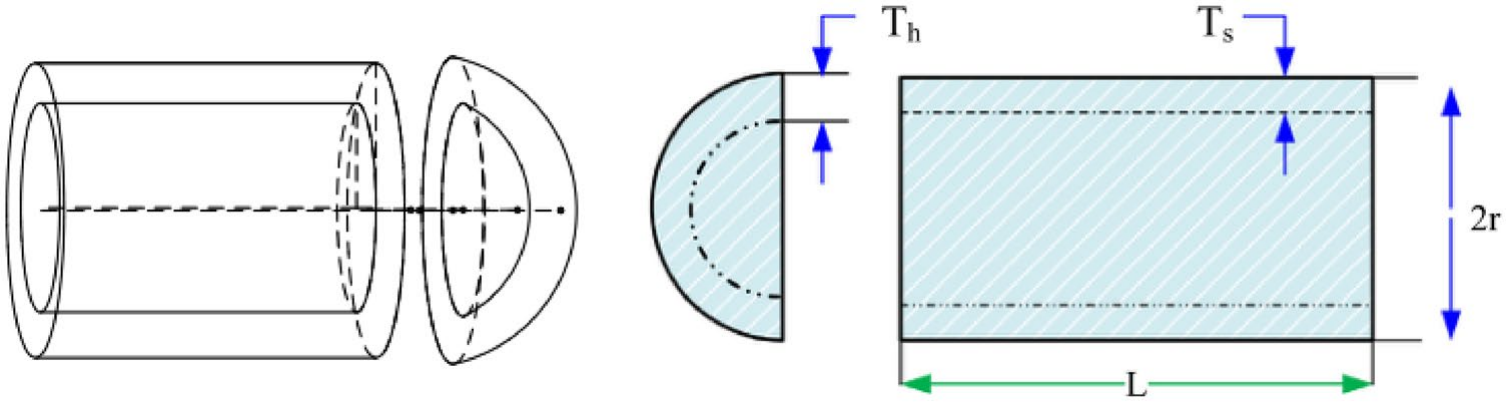
Pressure vessel design.

Kannan and Kramer^43^ proposed the pressure vessel design problem as shown in Fig. 38 to minimize the total cost, including the cost of material, forming, and welding. There are four design optimized objects: the thickness of the shell ${T}_{s}$; the thickness of the head ${T}_{h}$; the inner radius ${\text{R}}$; the length of the cylindrical section without considering the head ${\text{L}}$. The problem includes the objective function and constraints as follows:

$$\begin{aligned} & {\text{Consider}}\quad {\text{X}} = \left[ {x_{1} ,{ }x_{2} ,{ }x_{3} ,x_{4} \left] = \right[T_{s} ,{ }T_{h} ,{\text{ R}},{\text{ L}}} \right]; \\ & {\text{Objective function}}\quad f\left( x \right)_{ } = 0.6224x_{1} x_{3} x_{4} + 1.7781x_{2} x_{2}^{3} + 3.1661x_{1}^{2} x_{4} + 19.84x_{1}^{2} x_{3} \\ & {\text{Subject to}}\quad g_{1} \left( x \right) = - x_{1} + 0.0193x_{3} \le 0; \\ & \quad \quad \quad \quad \quad g_{2} \left( x \right) = - x_{3} + 0.00954x_{3} \le 0; \\ & \quad \quad \quad \quad \quad g_{3} \left( x \right) = - \prod x_{3}^{2} x_{4} - \frac{4}{3}\prod x_{3}^{3} + 1296000 \le 0; \\ & \quad \quad \quad \quad \quad g_{4} \left( x \right) = x_{4} - 240 \le 0; \\ & {\text{Variable range:}}\;0 \le x_{1} ,x_{2} \le 99;0 \le x_{3} ,x_{4} \le 200; \\ \end{aligned}$$

The results in Table 10 show that the NDWPSO algorithm obtains the lowest optimal cost with the same constraints and has the lowest standard deviation compared with other algorithms, which again proves the good performance of NDWPSO in terms of solution accuracy.

#### Three-bar truss design

**Figure 39:**
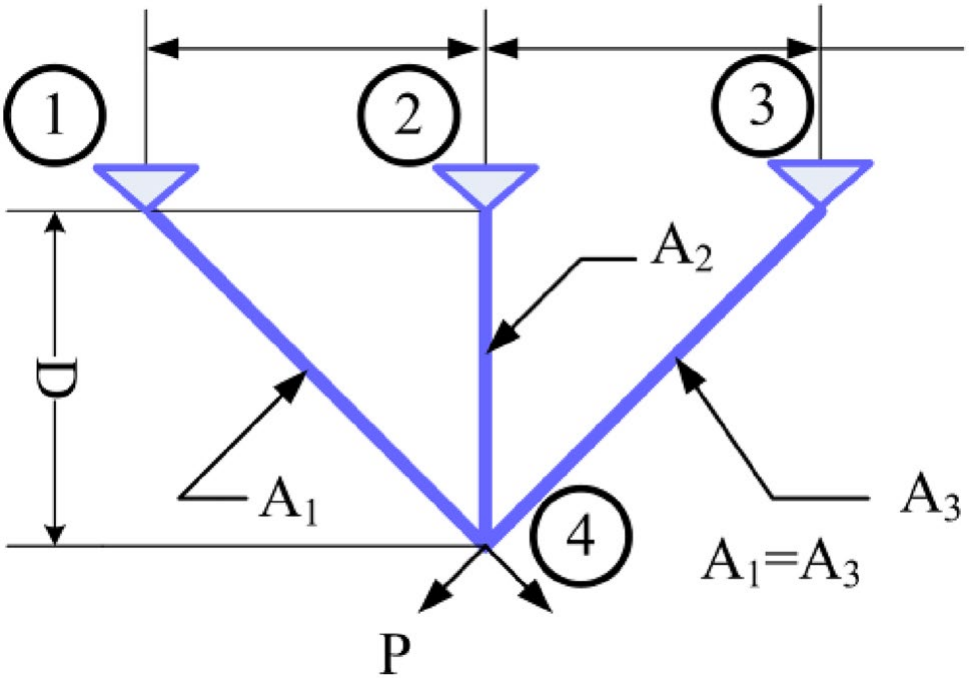
Three-bar truss design.

This structural design problem^44^ is one of the most widely-used case studies as shown in Fig. 39. There are two main design parameters: the area of the bar1 and 3 (${A}_{1}={A}_{3}$) and area of bar 2 (${A}_{2}$). The objective is to minimize the weight of the truss. This problem is subject to several constraints as well: stress, deflection, and buckling constraints. The problem is formulated as follows:

$$\begin{aligned} & {\text{Consider}}\quad {\text{X}} = \left[ {x_{1} ,x_{2} } \right] = \left[ {A_{1} ,A_{2} } \right] \\ & {\text{Objective function}} \quad f\left( x \right)_{ } = \left( {2\sqrt 2 x_{1} + x_{2} } \right) \times l \\ & {\text{Subject to}}\quad \quad g_{1} \left( x \right) = \frac{{\sqrt 2 x_{1} + x_{2} }}{{\sqrt 2 x_{1}^{2} + 2x_{1} x_{2} }}P - \sigma \le 0; \\ & \quad \quad \quad \quad \quad \quad g_{2} \left( x \right) = \frac{{x_{2} }}{{\sqrt 2 x_{1}^{2} + 2x_{1} x_{2} }}P - \sigma \le 0; \\ & \quad \quad \quad \quad \quad \quad g_{3} \left( x \right) = \frac{1}{{\sqrt 2 x_{2}^{ } + x_{1} }}P - \sigma \le 0; \\ & {\text{Variable range:}}\;0 \le x_{1} ,x_{2} \le 1; \\ & \quad \quad \quad \quad \quad \quad \;l = 100cm, P = 2KN/cm^{2} \sigma = 2KN/cm^{2} \\ \end{aligned}$$

From Table 11, NDWPSO obtains the best design solution in this engineering problem and has the smallest standard deviation of the result data. In summary, the NDWPSO can reveal very competitive results compared to other intelligent algorithms.

## Conclusions and future works

An improved algorithm named NDWPSO is proposed to enhance the solving speed and improve the computational accuracy at the same time. The improved NDWPSO algorithm incorporates the search ideas of other intelligent algorithms (DE, WOA). Besides, we also proposed some new hybrid strategies to adjust the distribution of algorithm parameters (such as the inertia weight parameter, the acceleration coefficients, the initialization scheme, the position updating equation, and so on).

23 classical benchmark functions: indefinite unimodal (f1-f7), indefinite multimodal (f8-f13), and fixed-dimensional multimodal(f14-f23) are applied to evaluate the effective line and feasibility of the NDWPSO algorithm. Firstly, NDWPSO is compared with PSO, CDWPSO, and SDWPSO. The simulation results can prove the exploitative, exploratory, and local optima avoidance of NDWPSO. Secondly, the NDWPSO algorithm is compared with 5 other intelligent algorithms (WOA, HHO, GWO, AOA, EO). The NDWPSO algorithm also has better performance than other intelligent algorithms. Finally, 3 classical engineering problems are applied to prove that the NDWPSO algorithm shows superior results compared to other algorithms for the constrained engineering optimization problems.

Although the proposed NDWPSO is superior in many computation aspects, there are still some limitations and further improvements are needed. The NDWPSO performs a limit initialize on each particle by the strategy of “elite opposition-based learning”, it takes more computation time before speed update. Besides, the” local optimal jump-out” strategy also brings some random process. How to reduce the random process and how to improve the limit initialize efficiency are the issues that need to be further discussed. In addition, in future work, researchers will try to apply the NDWPSO algorithm to wider fields to solve more complex and diverse optimization problems.

## Supplementary Information

Supplementary Information.

## Data Availability

The datasets used and/or analyzed during the current study available from the corresponding author on reasonable request.
