# Author Correction: A hybrid particle swarm optimization algorithm for solving engineering problem

**DOI:** 10.1038/s41598-024-75852-w

**Published:** 2024-10-22

**Authors:** Jinwei Qiao, Guangyuan Wang, Zhi Yang, Xiaochuan Luo, Jun Chen, Kan Li, Pengbo Liu

**Affiliations:** 1 School of Mechanical and Automotive Engineering, Qilu University of Technology (Shandong Academy of Sciences), Jinan, 250353 China; 2 Shandong Institute of Mechanical Design and Research, Jinan, 250353 China; 3 School of Information Science and Engineering, Northeastern University, Shenyang, 110819 China; 4 Fushun Supervision Inspection Institute for Special Equipment, Fushun, 113000 China

Correction to: *Scientific Reports*10.1038/s41598-024-59034-2, published online 10 April 2020

The original version of this Article contained an error in the Acknowledgements section, where the grant code for Key R&D plan was incorrect.

“This work was supported by Key R&D plan of Shandong Province, China (2021CXGC010207, 2023CXGC01020);”

now reads:

“This work was supported by Key R&D plan of Shandong Province, China (2021CXGC010207, 2023CXGC010208);”

The original Article has been corrected.

