# Supplementary material for: A hybrid particle swarm optimization algorithm for solving engineering problem
Source: Sci Rep. 2024 Apr 10;14:8357. doi: 10.1038/s41598-024-59034-2 (PMC11375002; doi:10.1038/s41598-024-59034-2)
Supplement: Supplementary file 1 — Supplementary Information. [file 41598_2024_59034_MOESM1_ESM.docx]

Supplementary：

**Supplementary Table S1. Description of unimodal benchmark functions(F1-F7)**

| **Function** | **Dim^2^** | **Range** | **Global opt.^1^** |
| --- | --- | --- | --- |
| $f_{1}=\sum_{i=1}^{n} x_{i}^{2}$ | 30,50,100 | [-100,100] | 0 |
| $f_{2}=\sum_{i=1}^{n} \left\vert x_{i} \right\vert+\prod_{i=1}^{n} \left\vert x_{i} \right\vert$ | 30,50,100 | [-10,10] | 0 |
| $f_{3}=\sum_{i=1}^{n} (\sum_{j=1}^{i} x_{j})^{2}$ | 30,50,100 | [-100,100] | 0 |
| $f_{4}=\max\left\{ \left. \left\vert x_{i} \right\vert, 1\leq i\leq n \right\} \right.$ | 30,50,100 | [-100,100] | 0 |
| $f_{5}=\sum_{i=1}^{n-1} \left[ {100\left( x_{i+1}-x_{i}^{2} \right)}^{2}+\left( x_{i}-1 \right)^{2} \right]$ | 30,50,100 | [-30,30] | 0 |
| $f_{6}=\sum_{i=1}^{n} {([x_{i}+0.5])}^{2}$ | 30,50,100 | [-100,100] | 0 |
| $f_{7}=\sum_{i=1}^{n} ix_{i}^{4}+random[0,1)$ | 30,50,100 | [-128,128] | 0 |

**^1^Global opt.**: **Global optimal solution. ^2^Dim: Dimension**

**Supplementary Table S2. Description of indefinite dimensional multimodal benchmark functions(F8-F13)**

| **Function** | **Dim** | **Range** | **Global opt.** |
| --- | --- | --- | --- |
| $f_{8}=\sum_{i=1}^{n} -x_{i}sin(\sqrt{\left\vert x_{i} \right\vert})$ | 30,50,100 | [-500,500] | -418.98$\times$ Dim |
| $f_{9}=\sum_{i=1}^{n} [x_{i}^{2}-10\cos\left( 2\pi x_{i} \right)+10]$ | 30,50,100 | [-5.12,5.12] | 0 |
| $f_{10}=-20\exp\left( -0.2\sqrt{\frac{1}{n}\sum_{i=1}^{n} x_{i}^{2}} \right)-\exp\left( \frac{1}{n}\sum_{i=1}^{n} \cos\left( 2\pi x_{i} \right) \right)+20+e$ | 30,50,100 | [-32,32] | 0 |
| $f_{11}=\frac{1}{4000}\sum_{i=1}^{n} x_{i}^{2}-\prod_{i=1}^{n} \cos\left( \frac{x_{i}}{\sqrt{i}} \right)+1$ | 30,50,100 | [-600,600] | 0 |
| $f_{12}=\frac{\pi}{n}\left\{ 10\sin\left( \pi y_{i} \right)+\sum_{i=1}^{n-1} \left( y_{i}-1 \right)^{2}\left[ 1+10{sin}^{2}\left( \pi y_{i+1} \right) \right]+\left( y_{n}-1 \right)^{2} \right\}+\sum_{i=1}^{n} u(x_{i},10,100,4)$ | 30,50,100 | [-50,50] | 0 |
| $f_{13}=0.1\left\{ {sin}^{2}\left( 3\pi x_{i} \right)+\sum_{i=1}^{n} \left( x_{i}-1 \right)^{2}\left[ 1+{sin}^{2}\left( 3\pi x_{i}+1 \right) \right]+\left( x_{n}-1 \right)^{2}\left[ 1+\sin^{2} \left( 2\pi x_{n} \right) \right] \right\}+\sum_{i=1}^{n} u(x_{i},5,100,4)$ | 30,50,100 | [-50,50] | 0 |

**Supplementary Table S3. Description of fixed-dimension multimodal benchmark functions(F14-F23)**

| **Function** | **Dim** | **Range** | **Global opt.** |
| --- | --- | --- | --- |
| $f_{14}={(\frac{1}{500}+\sum_{j=1}^{25} \frac{1}{j+\sum_{i=1}^{2} {(x_{i}-a_{ij})}^{6}})}^{-1}$ | 2 | [-65,65] | 1 |
| $f_{15}=\sum_{i=1}^{11} {[a_{i}-\frac{x_{1}(b_{i}^{2}+b_{i}x_{2})}{b_{i}^{2}+b_{i}x_{3}+x_{4}}]}^{2}$ | 4 | [-5,5] | 3.075e-04 |
| $f_{16}=4x_{1}^{2}-2.1x_{1}^{4}+\frac{1}{3}x_{1}^{6}+x_{1}x_{2}-4x_{2}^{2}+4x_{2}^{4}$ | 2 | [-5,5] | -1.0316 |
| $f_{17}=\left( x_{2}-\frac{5.1}{4\pi^{2}}x_{1}^{2}+\frac{5}{\pi}x_{1}-6 \right)^{2}+10\left( 1-\frac{1}{8\pi} \right)cosx_{1}+10$ | 2 | [-5,5] | 0.398 |
| $f_{18}=\left[ 1+\left( x_{1}+x_{2}+1 \right)^{2}\left( 19-14x_{1}+3x_{1}^{2}-14x_{2}+6x_{1}x_{2}+{3x}_{2}^{2} \right) \right]$  $\times[30+\left( 2x_{1}-3x_{2} \right)^{2}\times(18-32x_{1}+12x_{1}^{2}+48x_{2}-36x_{1}x_{2}+27x_{2}^{2})]$ | 2 | [-2,2] | 3 |
| $f_{19}=-\sum_{i=1}^{4} c_{i}exp(-\sum_{j=1}^{3} a_{ij}{(x_{j}-p_{ij})}^{2})$ | 3 | [1,3] | -3.86 |
| $f_{20}=-\sum_{i=1}^{4} c_{i}exp(-\sum_{j=1}^{6} a_{ij}{(x_{j}-p_{ij})}^{2})$ | 6 | [0,1] | -3.32 |
| $f_{21}=-\sum_{i=1}^{5} {[\left( X-a_{i} \right)\left( X-a_{i} \right)^{T}+c_{i}]}^{-1}$ | 4 | [0,10] | -10.1532 |
| $f_{22}=-\sum_{i=1}^{7} {[\left( X-a_{i} \right)\left( X-a_{i} \right)^{T}+c_{i}]}^{-1}$ | 4 | [0,10] | -10.4028 |
| $f_{23}=-\sum_{i=1}^{10} {[\left( X-a_{i} \right)\left( X-a_{i} \right)^{T}+c_{i}]}^{-1}$ | 4 | [0,10] | -10.5363 |
